# The diversity of cyanobacterial metabolism: genome analysis of multiple phototrophic microorganisms

**DOI:** 10.1186/1471-2164-13-56

**Published:** 2012-02-02

**Authors:** Christian Beck, Henning Knoop, Ilka M Axmann, Ralf Steuer

**Affiliations:** 1 Institute for Theoretical Biology, Charité-Universitätsmedizin, Invalidenstr. 43, D-10115 Berlin, Germany; 2 Institute for Theoretical Biology, Humboldt-University of Berlin, Invalidenstr. 43, D-10115 Berlin, Germany

## Abstract

**Background:**

Cyanobacteria are among the most abundant organisms on Earth and represent one of the oldest and most widespread clades known in modern phylogenetics. As the only known prokaryotes capable of oxygenic photosynthesis, cyanobacteria are considered to be a promising resource for renewable fuels and natural products. Our efforts to harness the sun's energy using cyanobacteria would greatly benefit from an increased understanding of the genomic diversity across multiple cyanobacterial strains. In this respect, the advent of novel sequencing techniques and the availability of several cyanobacterial genomes offers new opportunities for understanding microbial diversity and metabolic organization and evolution in diverse environments.

**Results:**

Here, we report a whole genome comparison of multiple phototrophic cyanobacteria. We describe genetic diversity found within cyanobacterial genomes, specifically with respect to metabolic functionality. Our results are based on pair-wise comparison of protein sequences and concomitant construction of clusters of likely ortholog genes. We differentiate between core, shared and unique genes and show that the majority of genes are associated with a single genome. In contrast, genes with metabolic function are strongly overrepresented within the core genome that is common to all considered strains. The analysis of metabolic diversity within core carbon metabolism reveals parts of the metabolic networks that are highly conserved, as well as highly fragmented pathways.

**Conclusions:**

Our results have direct implications for resource allocation and further sequencing projects. It can be extrapolated that the number of newly identified genes still significantly increases with increasing number of new sequenced genomes. Furthermore, genome analysis of multiple phototrophic strains allows us to obtain a detailed picture of metabolic diversity that can serve as a starting point for biotechnological applications and automated metabolic reconstructions.

## Background

Cyanobacteria are a unique phylogenetic group of bacteria and are the only known prokaryotes capable of oxygen-evolving photosynthesis. Cyanobacteria occupy diverse ecological niches and exhibit enormous diversity in terms of their habitats, physiology, morphology and metabolic capabilities. Due to their numerical abundance, most notably in marine environments, cyanobacteria have profound impact on almost all biochemical cycles that shape life on Earth. They are major players in global oxygen supply, carbon dioxide (CO_2_) sequestration, nitrogen fixation, as well as the primary phototrophic production of biomass. The latter capability, the utilization of atmospheric CO_2 _and sunlight for growth, has triggered renewed interest in the organization of cyanobacterial metabolism: Cyanobacteria are considered a promising resource for third generation biofuels and have attracted interest for a variety of related biotechnological applications [1-3]. However, while substantial knowledge is available for several model strains, the diversity of cyanobacterial metabolism remains poorly understood.

With the advent of novel sequencing techniques and the completion of several genome sequencing projects, a considerable number of complete cyanobacterial genome sequences are now available. This increasing number of sequenced genomes provides new opportunities for understanding microbial diversity and metabolic organization in diverse environments. Here, we report a whole genome comparison of multiple phototrophic cyanobacteria. Our focus is to describe the genetic diversity found within cyanobacterial genomes and to describe metabolic adaptations and diversity of several strains with different environmental background. Our work builds upon several previous studies on cyanobacterial genomic diversity and evolution [2-4]. For example, Raymond *et al*. [5] have previously compared five whole genome sequences from all groups of photosynthetic prokaryotes, with the aim to identify genes that play an essential role in phototrophy and to understand the advent and developement of photosynthesis. Their results showed that the genomes of the studied organisms resemble mosaics of genes with very different evolutionary histories and that orthologs common to all five genomes showed a distinct lack of unanimous support for any single phylogenetic topology. The importance of horizontal gene transfer (HGT) for cyanobacteria was later corroborated by the work of Zhaxybayeva *et al*. [6]. Shi and Falkowski [7] demonstrated an overall phylogenetic discordance among putative orthologous protein families from 13 genomes of cyanobacteria. The authors identified a core set of genes that was argued to be resistant to HGT and on which a robust organismal phylogeny can be constructed. Molecular synapomorphies, protein signatures that are present in an indicated group but not in other cyanobacteria or bacteria, were described by Gupta *et al*. [8,9] to further understand the evolutionary relationships between cyanobacteria. Mulkidjanian *et al*. (2006) [4] conducted a comparative analysis of 15 cyanobacterial genomes, with a focus on the origin of photosynthesis, and concluded that modern cyanobacteria inherited their photosynthetic apparatus from ancestral anaerobic phototrophs and not by lateral gene transfer from other phototrophic bacterial lineages. Recently, also several ocean sampling expeditions investigated microbial diversity in marine environments [10,11], again confirming substantial oceanic microbial diversity and considerable heterogeneity of microorganisms at the genomic level, specifically for Prochlorococcus, one of the most abundant genus of cyanobacteria.

Here, we augment the view on cyanobacterial genomic diversity with the identification and detailed analysis of putative orthologous genes across 16 cyanobacterial whole genome sequences. Our analysis is not restricted to a single genus of cyanobacteria but seeks to integrate representatives of cyanobacteria from almost all known environments. Unlike several previous studies, we do not aim to reconstruct evolutionary trajectories, but rather seek to describe differences and similarities in genome content. Our main focus is the role of metabolic genes of central carbon metabolism and hence metabolic functionality across diverse strains. The manuscript is organized as follows: First, we define clusters of likely ortholog genes, denoted as CLOGs, based on pair-wise comparison of protein sequences. Subsequently, we investigate the core and pan-genome of cyanobacterial strains and discuss codon usage analysis, as well as gene sharing and phylogenetic congruence. In the final three sections, we focus on the diversity of cyanobacterial metabolism and discuss how specific enzymes, and hence metabolic pathways and capabilities are distributed across selected cyanobacterial strains.

## Results and Discussion

### Genome analysis and ortholog cluster

Starting point of our analysis are the genome sequences of 16 selected cyanobacteria, as obtained from GeneBank (http://www.ncbi.nlm.nih.gov/genbank). The chosen strains are not restricted to a single genus but were selected to represent the known genomic and metabolic diversity found in the cyanobacterial phylum, including eight marine and eight freshwater strains. The selected cyanobacterial strains include the model organisms *Synechocystis sp*. PCC 6803, *Synechococcus elongatus *PCC 7492 and *Cyanothece sp*. ATCC 51142, several nitrogen-fixing cyanobacteria (diazotrophs), as well as two thermophiles originally isolated from hot-spring environments. Details on the choice of strains are provided in Methods and a summary of the properties of the selected strains is given in Table 1. A phylogenetic tree based on 16S rRNA is shown and discussed further below.

**Table 1:** Selected cyanobacterial strains.

				Genome			DNA	Nitrogen			
		**Abbrev**.	Type	size (Mb)	G+C	Genes	coding (%)	fixation	Habitat	**Arrang**.	**Subsect**.
***Acaryochloris marina MBIC11017***		Aca11017	*β*	8.36	46.96	8488	83.26	-	M	S	I
***Cyanothece sp***. **ATCC 51142**		Cyn51142	*β*	5.46	37.94	5354	86.80	•	M	S	I
***Cyanothece sp. PCC 8801***		Cyn8801	*β*	4.79	39.76	4615	84.85	•	F	S	I
***Gloeobacter violaceus PCC 7421***		Glo7421	*β*	4.66	62.00	4490	89.36	-	F	S	I
***Microcystis aeruginosa NIES-843***		Mic843	*β*	5.84	42.33	6360	81.43	-	F	S	I
***Nostoc sp***. **PCC 7120**		Nos7120	*β*	7.21	41.27	6222	82.50	•	F	F	IV
***Prochlorococcus***		ProMED4	*α*	1.66	30.80	1766	88.42	-	M	S	I
***marinus *MED4**											
***Prochlorococcus***		Pro9211	*α*	1.69	38.01	1901	90.12	-	M	S	I
***marinus *MIT 9211**											
***Prochlorococcus***		Pro9215	*α*	1.74	31.15	2059	89.62	-	M	S	I
***marinus *MIT 9215**											
***Synechococcus sp***.		SycJA23	*β*	3.05	58.45	2947	85.48	•	F/T	S	I
**JA-2-3B'a(2-13)**											
***Synechococcus***		Syc7002	*β*	3.41	49.19	3237	87.64	-	M	S	I
***sp*. PCC 7002**											
***Synechococcus***		Syc7803	*α*	2.37	60.24	2591	93.39	-	M	S	I
***sp*. WH7803**											
***Synechococcus***		Syc7942	*β*	2.80	55.43	2719	89.21	-	F	S	I
***elongatus *PCC 7942**											
***Synechocystis***		Syn6803	*β*	3.57	47.37	3628	86.74	-	F	S	I
***sp*. PCC 6803**											
***Thermosynechococcus***		ThermoBP1	*β*	2.59	53.92	2555	89.99	-	F/T	S	I
***elongatus *BP-1**											
***Trichodesmium***		Trich101	*β*	7.75	34.14	5156	60.11	•	M	F	III
***erythraeum *IMS101**											

A summary of the 16 different cyanobacterial strains considered in this study. Given is the respective abbreviation, type of the cyanobacterial species which is based on their type of RuBisCO [41], genome size (Mb), C+G content, the number of identified genes and the percentage of coding DNA according to IMG database [42], the ability of the strain to fixate nirtrogen, habitat and cell arrangement. Within the column for habitat marine strains are marked by an M, fresh water by an F, thermophile strains are marked by a T. Cell arrangement is subdivided in single cells (S) and filamentous cell arrangement (F). The division of the strains into different subsections is according to [43].

To investigate genomic diversity, we aim to identify groups of ortholog genes, based on a pair-wise all-against-all comparison of identified protein sequences. Two protein sequences are regarded as likely orthologs if the reciprocal comparison results in a bidirectional hit rate (BHR) larger than a given threshold. Subsequently, likely orthologs were assigned to clusters by merging ortholog pairs. Clusters of likely ortholog genes were then checked for consistency and, if applicable, split into separate clusters. In this way, gene pairs within one cluster that exhibit a BHR below a given threshold are avoided. We restrict the analysis to the chromosome, plasmids are not considered. Details of the algorithm are given in Material and Methods. Our approach follows earlier approaches to detect putative orthologs across several genome sequences [4,5,12-17]. However, we adopt rather stringent criteria to avoid inclusion of erroneous non-ortholog pairs, at the expense of potentially underestimating the number of true orthologs.

**Figure 1:**
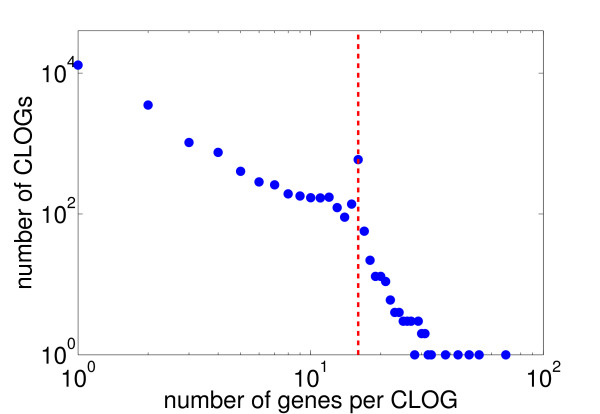
**Number of genes per cluster of likely ortholog genes (CLOGs)**. The majority of CLOGs consist of only one gene. CLOGs with 16 genes, indicated by the vertical line, are overrepresented. Only few clusters consist of more than 16 genes and almost no cluster consists of more than 32 genes.

Our algorithm results in 21238 distinct clusters of likely ortholog genes (CLOGs), distributed across all 16 strains (data in Additional File 1). Figure 1 shows a histogram of the number of assigned genes per CLOG. The majority of clusters, almost 60%, consists of a single gene (singletons), whereas only a small number of clusters have more than 30 or 40 members. CLOGs with exactly 16 members are overrepresented, indictated in Figure 1 by a vertical line. Overall, the distribution differs slightly from the results provided in the COG database [12,18]. Therein, considering only the two cyanobacterial strains (Syn6803 and Nos7120) included in the database, clusters of ortholog genes tend to be comprised of more genes, often including multiple genes from the same strain.

**Figure 2:**
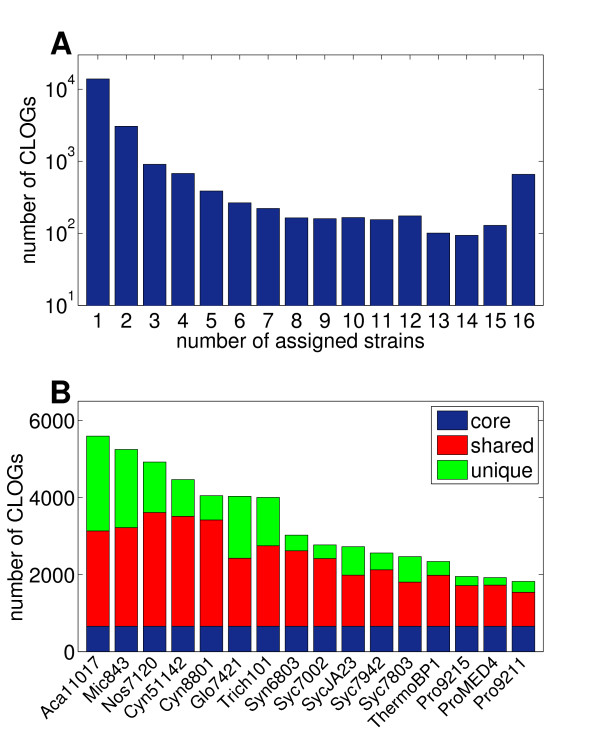
**Distribution of CLOGs across 16 cyanobacterial genomes**. **A **- A histogram of the number of assigned strains to each CLOG. We distinguish between core CLOGs (660 CLOGs, assigned to all 16 strains), shared CLOGs (6668 CLOGs, assigned to 2-15 strains), and unique CLOGs (13910 CLOGs, assigned to a unique strain). **B **- Number of CLOGs assigned to each strain, highlighting the contribution of core, shared, and unique CLOGs.

To obtain insight into the organization of the cyanobacterial genomic diversity, each CLOG is assigned to a cyanobacterial strain if one or more member of a CLOG is present in the respective genome. Figure 2A shows a histogram of the number of CLOGs as a function of the number of associated strains. We can distinguish between core genes (660 CLOGs), those that are assigned to all 16 strains, shared genes (6668 CLOGs), those that are found in more than one but not in all strains, and unique genes (13910 CLOGs) that have no likely ortholog in any other of the 15 genome sequences. Figure 2B shows the number of CLOGs assigned to each cyanbacterial species, highlighting the contribution of core, shared, and unique CLOGs. The data is provided as Additional File 2.

We observe that the majority of ortholog clusters is associated with a single genome, and therefore represent unique genes with no likely ortholog in any other of the considered strains. The number of CLOGs shared among two or more genomes then quickly drops. We note that the scale in Figure 2A is logarithmic. However, a significant number of CLOGs is again assigned to the core genome. Clusters of likely ortholog genes that are present in all 16 cyanobacterial genomes are more frequent than clusters that are only shared between any given number, but not all, strains. The set of core CLOGs is in good agreement with the results reported in Mulkidjanian *et al*. (2006) [4]. Specifically, when using Syn6803 as a reference, almost all genes assigned to a core CLOG (*>*90%) in our analysis are likewise a member of a core cyanobacterial clusters identified by Mulkidjanian *et al*. [4]. Our results are also in good qualitative agreement with several previous studies on other bacterial lineages. For example, Hogg *et al*. [14] observed a similar distribution for 12 sequenced strains of *Haemophilus influenzae*. Extending the pan-genome concept to higher taxonomic units, Lapierre and Gogarten [19] report a shared core genome of approximately 250 genes across more than 500 sequenced bacterial genomes. In both cases, corresponding to the results shown in Figure 2, a U-shaped distribution was observed, such that unique and core genes are overrepresented compared to any single set of genes assigned to a finite number of genome sequences.

### The cyanobacterial core- and pan-genome

Whole genome comparisons offer the possibility to extrapolate the observed results beyond the number of strains explicitly considered in the comparison. In this respect, pan-genome analysis has recently emerged as a novel approach to estimate the size of the gene repertoire accessible to any given species [20]. A number of recent studies have found consistently that the number of genes accessible to a bacterial species is usually orders of magnitude larger than the number of genes contained in the genome of any single organism. These results have a direct implication for resource allocation and whole-genome sequencing projects, as they can potentially predict how many new genes are identified every time a new genome of the species of interest is sequenced.

**Figure 3:**
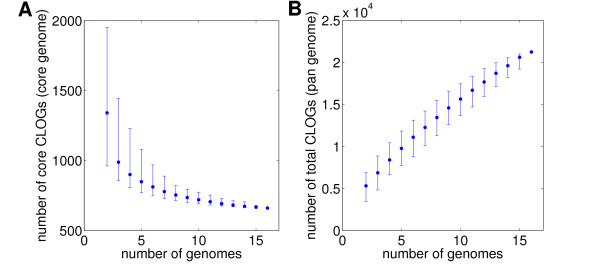
**The cyanobacterial pan- and core-genome**. Estimated size of core- (**A**) and pan- (**B**) genome with increasing number of considered genomes. To avoid dependency on strain order, the 16 cyanobacterial strains were arranged in random order. At each step, we recalculated the number of core CLOGs (CLOGs assigned to all strains included as yet) and pan CLOGs (all CLOGs as yet found in at least one of the included strains) genome. This procedure was repeated 1000 times, the median across all iterations is shown. The errorbars represent the 0.1 and 0.9 quantiles estimated from 1000 iterations.

Figure 3 shows the size of the cyanobacterial core- and pan-genome estimated from the 16 strains considered here. The total pan-genome of all 16 strains encompasses more than 2·10^4 ^ortholog clusters and the increase as a function of the number of genomes does not show substantial flattening of the curve (Figure 3B). With each newly included genome still more than approximately 500 novel ortholog clusters are added to the pan-genome. Given these rarefaction curves, it must be expected that sequencing of further cyanobacterial strains will still result in the discovery of a high number of as yet unknown genes, even when the number of sequenced genomes goes significantly beyond the number sequenced as yet. The results shown in Figures 2 and 3 give rise to two questions. First, what is the size of the total cyanobacterial pan-genome? And, second, what is the functional and evolutionary difference, if any, between the core, shared and unique genes? Both questions have been addressed in the recent literature but cannot be resolved with any certainty yet.

For the size of the bacterial pan-genome, divergent results have been obtained for different species. Hogg *et al*. [14], reported a finite pan-genome for *Haemophilus influenzae*, extrapolating from 12 whole genome sequences, while results for *Streptococcus agalactiae *indicate an infinite asymptotic pan-genome [21]. These results may indeed reflect differences in ecologial niches and evolutionary history. However, a fundamental objection to mathematical extrapolation has been raised recently [17]. As argued by Kislyuk *et al*. [17] such extrapolation estimates are likely to be spurious because they depend on the estimation of the occurence of extremely rare genes and genomes, respectively, which are problematic to estimate precisely because they are rare. Therefore, we do not give any estimate for the total cyanobacterial pan-genome. Nonetheless, we consider several key findings to be valid: There is a core genome that is shared between all 16 cyanobacterial strains considered here. The asymptotic size of the core genome when exptrapolated to all cyanobacterial strains is currently unknown. Furthermore, there is no indication that the cyanobacterial pan-genome is closed. Therefore, the results shown in Figure 3 provide a strong incentive for further genome sequencing even of closely related strains.

A second issue relates to the possible functional and evolutionary differences between shared, core and unique genes. Common to all recent studies is that the number of unique genes, and those that are only shared between a small number of genomes, represents a rather large proportion of the total gene repertoire [22]. A variety of hypotheses with respect to the origin of such a distribution have been put forward. For example, core genes are often assumed to be predominantely related to housekeeping functions [22]. Unique genes, on the other hand, may be characteristic to specific environments and are assumed to be subject to extensive HGT [6,7].

We tested this assertion by comparing the annotation obtained from gene ontology (GO) database [23]. An analysis of the GO annotation of core CLOGs reveals a significant enrichment of genes related to "translation" (p-value *<*1·10^-30^), "DNA repair" (p-value *<*1·10^-4^), "gene expression" (p-value *<*1.8·10^-7^), "RNA processing/modification" (p-value *<*1·10^-5^), diverse transporting processes (p-values *<*1·10^-4^), as well as several metabolic and biosynthetic processes (p-values *<*1·10^-5^). Genes of the unique CLOGs are enriched with the annotation "defence response" (p-value *<*1.6·10^-5^), "DNA integration" (p-value *<*8.2·10^-5^), and are in particular enriched in annotations of regulatory processes. The latter may implicate a conservation of a functional core, such as metabolism and gene expression machinery, whereras regulatory properties and interactions are more specific to diverse environments. A complete list of the enriched GO terms is provided in Additional File 3. Algorithmic details are given in the Methods. We also need to point out a possible bias due to a significant enrichment of GO annotated genes in the core CLOGs (p *<*9·10^-210 ^with Fisher's exact test), whereas genes associated with unique clusters are more likely to have no GO annotation. This imbalance can be explained by the fact that GO annotations are mainly based on BLAST searches in other species and unique genes can therefore be expected to have fewer matches. Furthermore, a significant fraction of unique genes may also be due to annotation errors or be non-functional as part of an ongoing process of genome reduction and pseudogenization [22].

### Codon usage analysis

To further elucidate the difference between core and pan-genome, we compared the codon usage of the respective CLOGs across the 16 cyanobacterial genomes. To this end, the relative abundance of each nucleotide triplet for each amino acid was estimated and we calculated $d_{g1,g2}^{2}$ as the sum of squared differences in codon usage between any two groups of genes, *g*1 and *g*2, respectively. See Methods for algorithmic details. We found considerable differences in codon frequencies for the set of core genes between different strains, indicating adaptation of the codon usage to the respective strain. For all 16 strains, the codon usage frequency was found to be significantly different between the set of core and unique genes. To quantify the difference in codon usage between the set of of core and unique genes, we use the ratio *r_x _*of the sum of squared differences in codon usage between core/core and core/unique genes for each strain. This ratio ranges from *r_x _*= 2 for Pro9215 up to *r_x _*= 24 for Syn6803 and Mic843. A table with detailed information is provided as Additional File 4. The difference in codon usage between core and unique genes within any single strain typically exceeds the differences between core genes, as well as between unique genes, across different strains. The difference in codon usage between core and unique genes is lowest in the rather small genomes of the three Prochlorococcus strains Pro9215, ProMED4, and Pro9211.

### Gene sharing and phylogenetic congruence

**Figure 4:**
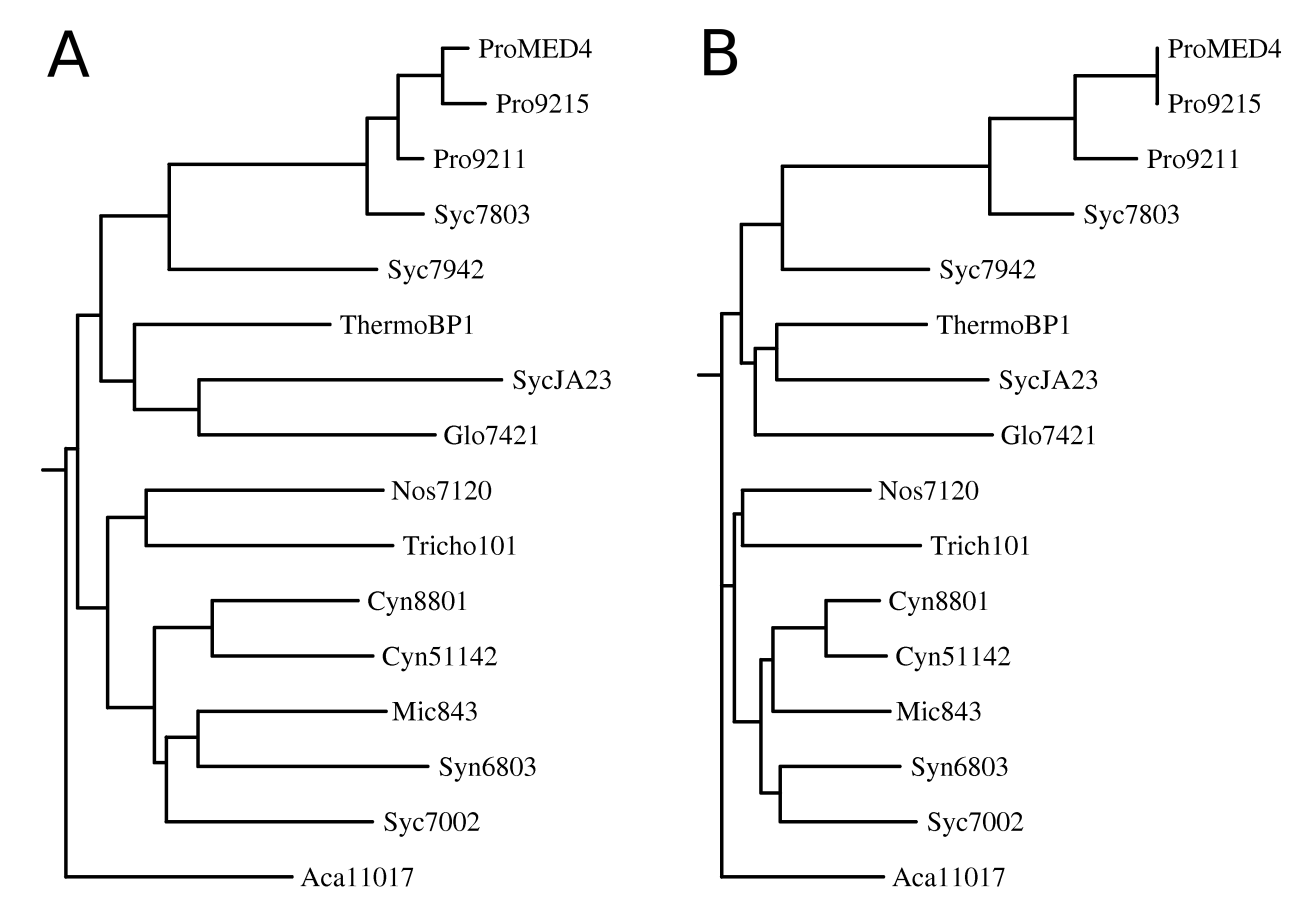
**Phylogenetic analysis of cyanobacterial strains**. **A **- Phylogenetic tree based on 16S rRNA comparison. **B **- Phylogenetic tree based on the number of shared CLOGs in common for pairs of strains. For the left figure, a tree in newick format was extracted from the Ribosomal Database Project web site (http://rdp.cme.msu.edu/) by selecting the 16 strains considered in this study and exporting the tree built with TREE BUILDER. The tree was plotted with DRAWGRAM of the phylogeny inference package (PHYLIP). For the right figure, a similarity matrix was calculated, such that the similarity between two strains was defined by the number of shared CLOGs divided by number of total CLOGs assigned to both strains. Subsequently, all entries in the matrix are substracted from the maximal entry.

We are interested in the relationships between cyanobacterial species based on gene sharing, as compared to 16S rRNA analysis. Figure 4A shows a phylogenetic tree obtained from 16S rRNA, using PHYLIP (phylogeny inference package version 3.69) by Felsenstein [24]. Several options to estimate similarity based on assignment of CLOGs are available. Here, we use a simple measure based on the number of CLOGs common to two strains divided by the total number of CLOGs associated with both strains combined. The respective distance tree is shown in Figure 4B. Both trees exhibit a high degree of similarity, with only minor topological differences. In both cases, the Prochlorococcus strains form the closest related cluster. We note that we do not consider phylogenetic trees of individual gene families, where a higher degree of phylogenetic discordance must be expected [7]. Likewise any estimate of distance based on shared CLOGs is likely biased by genome size, which again reflects evolutionary distance as determined by 16S rRNA analysis. Table 2 gives a pair-wise comparison of shared CLOGs between all 16 cyanobacterial strains. The table confirms the close association of the three Prochlorococcus strains with Syc7803 with respect to shared genes.

**Table 2:** Pair-wise comparison of cyanobacterial strains.

	Cyn51142	Cyn8801	Glo7421	Mic843	Nos7120	ProMED4	Pro9211	Pro9215	SycJA23	Syc7002	Syc7803	Syc7942	Syn6803	ThermoBP1	Trich101
**Aca11017**	2023	1952	1574	1841	2167	1016	1018	997	1454	1736	1228	1602	1726	1572	1765
**5592**															
	**Cyn51142**	2721	1561	2285	2379	988	1008	979	1481	1903	1238	1625	2049	1557	1980
	**4465**														
		**Cyn8801**	1537	2301	2265	983	1004	970	1474	1871	1208	1622	2043	1537	1916
		**4048**													
			**Glo7421**	1546	1741	868	892	853	1333	1334	1037	1278	1385	1269	1378
			**4032**												
				**Mic843**	2216	988	1000	963	1450	1788	1186	1626	1951	1517	1863
				**5247**											
					**Nos7120**	989	1000	974	1596	1911	1228	1671	1906	1614	2079
					**4920**										
						**ProMED4**	1334	1616	904	973	1277	1022	972	924	974
						**1922**									
							**Pro9211**	1309	908	983	1340	1040	986	944	992
							**1825**								
								**Pro9215**	888	962	1285	998	961	910	964
								**1951**							
									**SycJA23**	1380	1055	1345	1379	1364	1418
									**2725**						
										**Syc7002**	1169	1547	1812	1459	1635
										**2771**					
											**Syc7803**	1265	1169	1109	1182
											**2467**				
												**Syc7942**	1581	1441	1491
												**2561**			
													**Syn6803**	1490	1644
													**3024**		
														**ThermoBP1**	1450
														**2340**	
															**Trich 101**
															**4004**

A table of shared CLOGs. Each entry shows the number of CLOGs associated with both strains. The left-most column in each row gives the total number of CLOGs associated with each strain.

### The metabolic network is highly conserved

Going beyond pan-genome analysis, we are particularly interested in the organization and diversity of cyanobacterial metabolism. To identify those CLOGs that can be associated with metabolic function, we utilize the Enzyme Commison (EC) number of each gene, as obtained from the KEGG database: A CLOG is regarded as metabolic if the respective set of orthologous genes can be assigned to one or more EC numbers associated with a specific enzymatic activity. We note that due to the hierarchical classification scheme, this assignment may also include broad enzymatic categories, as well as a limited number of non-metabolic enzymatic functions. See Methods for algorithmic details and some caveat.

**Figure 5:**
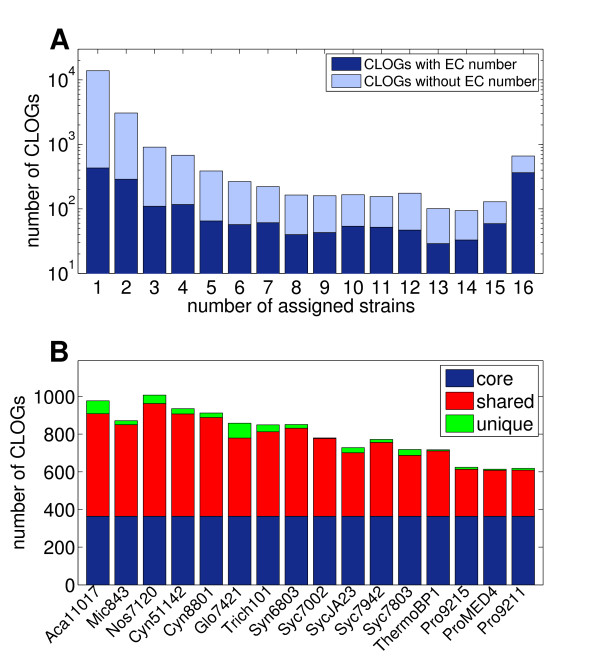
**Distribution of metabolic CLOGs across cyanobacterial genomes**. **A **- A histogram of the number of assigned genes to CLOGs, highlighting contributions from CLOGs with and without assigned EC number. CLOGs with assigned EC number are highly prevalent among the core CLOGs. We note that the scale is logarithmic. **B **- Number of CLOGs with dedicated EC number assigned for each strain, distinguishing between contributions from core, shared, and unique CLOGs.

**Figure 6:**
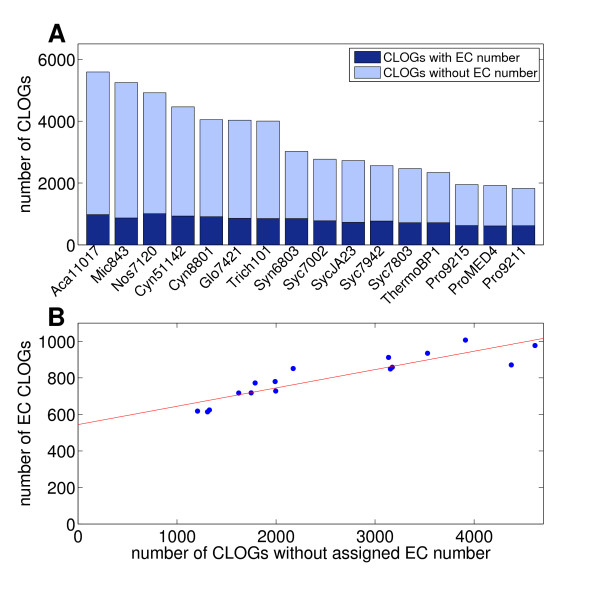
**Comparison of CLOGs with and without assigned EC numbers**. **A **- A bar plot of the number of CLOGs with and without assigned EC number in all 16 strains. **B **- Number of CLOGs with assigned EC number compared to CLOGs without assigned EC number in the strains of different size. The red line indicates a least square regression line to highlight the tendency of the data. The data shows a weak correlation of size (total number of CLOGs) and number of CLOGs with assigned EC numbers.

Using the set of CLOGs described above, 1851 CLOGs of the 21238 can be regarded as metabolic. We note that due to bifunctional enzymes or inconsistent and erroneous annotation CLOGs may be assigned to more than one metabolic function. However, in our case only 66 CLOGs (out of 1851) are assigned to more than one EC number, with a total of 759 distinct EC numbers assigned across all clusters. These results indicate that inconsistent annotation does not significantly constrain our analysis, even without prior filtering or manual curation. Figure 5 shows the distribution of metabolic CLOGs across the unique, shared and core genome. CLOGs assigned to metabolic function are highly overrepresented within the set that is common to all 16 cyanobacterial strains, with about 55% of all core CLOGs associated with metabolic function. Obviously, cellular metabolism, defined here as genes assigned to enzymatic function, constitutes a large fraction of the core genome. Figure 6 gives the percentage of CLOGs assigned to enzymatic function across all 16 strains considered in this study. The number of enzymatic CLOGs increases linearly with the number of total CLOGs assigned to each strain, with an offset of about 500 core enzymatic CLOGs. However, the correlation between number of enzymatic and total CLOGs is rather weak and dominated by the contribution from enzymatic core CLOGs. A further analysis of the respective pathways and enzyme classes associated to CLOGs revealed no obvious difference between unique and core genes, that is, no particular enzymatic category or pathway was strongly overrepresented in either class. Nonetheless, a number of core pathways can be identified that are common to all 16 cyanobacterial strains. Among the highly conserved pathways are the Calvin Benson cycle, the oxidative pentose phosphate pathway, nucleotide synthesis, and amino acids synthesis. However, with respect to the latter, a number of phosphatases and transaminases are not annotated in several strains.

### The diversity of cyanobacterial metabolism

**Figure 7:**
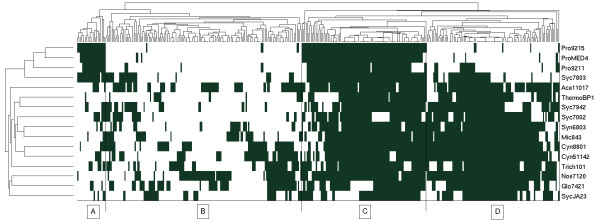
**Diversity of cyanobacterial metabolism**. Shown is a clustered heatmap of the association between the 378 shared EC numbers and the 16 cyanobacterial strains. The clustering of cyanobacterial strains is in good agreement with the results shown in Figure 4. With respect to annotated EC numbers, four broad categories can be distinguished: Category A corresponds to EC numbers predominantely annotated for the three Prochlorococcus strains and Syc7803. Category B corresponds to EC numbers associated with only a small number of strains. Category C covers EC numbers that are associated with almost all strains. Category D corresponds to those EC numbers that are associated with most strains, but are less prevalent in the Prochlorococcus strains and Syc7803. The categories are identified by visiual inspection of the clustered heatmap.

A multifaceted picture is obtained, if we look how specific enzymes, and hence metabolic capabilities, are distributed across the 16 cyanobacterial strains. To this end, we first limit the analysis to shared EC numbers. Of the total of 759 distinct EC numbers, assigned across all clusters, a subset of 378 EC numbers is associated with more than one, but less than 16 strains. Figure 7 provides a clustered heatmap of the association between these 378 shared EC numbers and the 16 cyanobacterial strains. EC numbers were clustered using the matlab function clustergram with distance 'hamming'. Overall, we can distinguish between four broad categories: First, shared EC numbers that are predominantely annotated with the Prochlorococcus strains, Pro9215, ProMed4, Pro9211 and Syc7803 (Cluster A in Figure 7). Second, shared EC numbers that are only annotated to a small number of strains (Cluster B in Figure 7). Third, shared EC numbers that are annotated to a large number of strains (Cluster C in Figure 7), and, fourth, shared EC numbers that are annotated to almost all strains, except the three Prochlorococcus strains and Syc7803. We note that Figure 7 again underscores the similarity between the three Prochlorococcus strains and Syc7803 that is already apparent in Figure 4 and Table 2.

Investigating the associations more closely confirms differences between the four groups. Exclusively associated with the four strains ProMED4, Pro9211, Pro9215 and Syc7803 are the two EC Numbers EC 1.1.5.4, a malate:quinone oxidoreductase involved in the TCA cycle and EC 2.5.1.48, a cystathionine gamma-synthase which catalyzes one of the essential steps in the synthesis of the amino acid L-methionine. For most other strains the synthesis pathway of methionine is unclear. No EC numbers are solely associated with the three Prochlorococcus strains, but no other strain. Vice versa, group D contains a set of 11 EC numbers that are associated with all strains, except the three Prochlorococcus strains ProMED4, Pro9211, Pro9215. This set includes EC 1.7.7.1, a nitrite reductase, and EC 1.7.7.2, a nitrate reductase. Indeed, it was widely assumed that nitrate is unavailable for Prochlorococcus because none of the sequenced laboratory strains contain the respective gene for nitrate utilization [25]. However, this view was recently challenged as metagenomic sequence data revealed that several Prochlorococcus ecotypes may contain nitrate reductase genes [26]. EC numbers that are not annotated for the Prochlorococcus strains and Syc7803, include several enzymatic activities of central metabolism, such as EC 1.1.1.38 (malic enzyme), EC 1.1.1.94 (glycerol-3-phosphate dehydrogenase), and EC 3.1.3.11 (fructose-1,6-bisphosphatase). A comprehensive list of differences in EC annotations between group A and group D is given as Additional File 5. A detailed discussion of genes involved in central metabolism is provided below.

### Cyanobacterial storage metabolism

As phototrophic organisms, most cyanobacteria rely on indigenous compounds that act as storage and allow to maintain cellular function during the night and in the absence of light. The most prevalent storage compound in cyanobacteria is glycogen, a branched polymer synthesized from glucose-6-phosphate. Glycogen is assumed to be accumulated during the day and mobilized during periods of darkness.

Accumulation of glycogen is also relevant under conditions of nitrogen limitation. All 16 strains considered here possess the necessary enzymes for glycogen synthesis and mobilization. In particular, a CLOG that is annotated with the enzyme AGP (EC 2.7.7.27) belongs to the core genome and is associated with all 16 strains. Likewise, the enzymes GS (EC 2.4.1.21) and GBE (EC 2.4.1.18) are annotated for all 16 cyanobacterial strains. The respective enzymes are associated with different CLOGs and are therefore not necessarily orthologs within all strains. The enzyme responsible for glycogen mobilization GP (EC 2.4.1.1) is again associated with all 16 strains, albeit not as a single CLOG. See Table 3 for an overview. We note that in all cases where an enzyme is associated with more than one CLOG, there usually is a dominant CLOG associated to almost all strains and a small number of secondary CLOGs whose members are annotated with the same enzyme. On closer inspection, this distinction is not an artifact of the clustering algorithm, but is supported by pair-wise comparisons of the respective sequences.

**Table 3:** Cyanobacterial storage metabolism.

	*Glycogen*										*Cyanophycin*				***Poly-β-hydroxybutyrate***			
	AGP	GS		GBE			GP				CphA			CphB	PhaA	PhaB	PhaC	PhaE
**Aca11017**	••	•	•	•	-	-	•	-	-	-	-	-	-	-	•	-	-	-
**Cyn51142**	••	•	•	•	•	•	••	-	-	-	•	-	-	•	-	-	-	-
**Cyn8801**	•	•	•	•	-	•	••	-	-	-	•	-	-	•	-	-	-	-
**Glo7421**	•	•	-	•	•	-	••	•	-	•	•	-	-	•	-	-	-	-
**Mic843**	•	•	•	•	-	-	•	-	-	-	•	-	-	•	•	•	•	•
**Nos7120**	•	•	•	•	•	-	-	•	•	-	•	•	-	•	-	-	-	-
**ProMed4**	•	•	-	•	-	-	•	-	-	-	-	-	-	-	-	-	-	-
**Pro9211**	•	•	-	•	-	-	•	-	-	-	-	-	-	-	-	-	-	-
**Pro9215**	•	•	-	•	-	-	•	-	-	-	-	-	-	-	-	-	-	-
**SycJA23**	•	•	•	•	-	-	-	••	-	-	•	•	-	•	-	-	-	-
**Syc7002**	•	•	•	•	-	-	•	•	-	-	•	-	-	•	-	-	-	-
**Syc7803**	•	•	-	•	-	-	•	-	-	-	-	-	-	-	-	-	-	-
**Syc7942**	•	•	-	•	-	-	•	-	-	-	-	-	(•)	-	-	-	-	-
**Syn6803**	•	•	•	•	-	-	••	-	-	-	•	-	-	•	•	•	•	•
**ThermoBP1**	•	•	-	•	-	-	••	-	•	-	•	-	-	•	-	-	-	-
**Trich101**	•	•	-	•	-	-	••	-	-	-	•	-	-	•	-	-	-	-

A summary of the genes involved in the synthesis and mobilization of the different storage compounds within the 16 cyanobactrial strains. Black dots represent the number of genes associated with a CLOG assigned to the specific enzymatic function. Columns correspond to different CLOGs that may be assigned to the same enzymatic activity. Abbreviations are: AGP: ADP glucose pyrophosphorylase (EC 2.7.7.27), GS: Glycogen synthase (EC 2.4.1.21), GBE: Glycogen branching enzyme (EC 2.4.1.18), GP: Glycogen phosphorylase (EC 2.4.1.1), CphA: Cyanophycin synthetase (EC 6.-.-.-), CphB: Cyanophycinase (EC 3.4.15.6), PhaA: PHA-specific *β*-ketothiolase/Acetyl-CoA acetyltransferase (EC 2.3.1.9), PhaB: PHA-specific acetoacetyl-CoA reductase (EC 1.1.1.36), PhaC/E: Poly (3-hydroxyalkanoate) synthase (EC 2.3.1.-).

Compared to glycogen, other storage compounds are less ubiquitous. Nonetheless, for the majority of strains, the enzymes for cyanophycin synthesis and mobilization are annotated. Cyanophycin is a polymer composed of aspartate and arginine and serves as a source of nitrogen and carbon in several cyanobacteria. As in the case for glycogen, the enzyme for cyanophycin synthesis (CphA, EC 6.3.2.29/30, see Knoop *et al*. [27]) is associated with several CLOGs, whereas the enzyme for cyanophycin mobilization (CphB, EC 3.4.15.6) is confined to a single CLOG. Both enzymes always occur together. That is, no strain is annotated only with synthesis or mobilization, with a single exception for Syc7803. However, in this case, the associated singleton CLOG is likely to be a erroneous annotation. The respective gene is annotated as a putative cyanophycin synthetase in CyanoBase, but the similarity to other known genes encoding cyanophycin synthetase is low.

Less prevalent than cyanophycin is the utilization of poly-beta-hydroxybutyrate (PHB) as a storage for carbon. PHB is a nontoxic biodegradable polyester of biotechnological importance, whose production by genetically engineered cyanobacteria was discussed recently [28]. Among the 16 strains considered here, the corresponding enzymes for synthesis of PHB are annotated only for *Synechosystis sp*. PCC 6803 (Syn6803) and *Microcystis aeruginosa *NIES-843 (Mic843). We note that although the strain Aca11017 is also associated with the CLOG annotated with PhaA (PHA-specific *β*-ketothiolase/Acetyl-CoA acetyltransferase, EC 2.3.1.9), the strain lacks the remaining steps for PHB synthesis. The respective gene in Aca11017 is a close variant of PhaA that is not specific for PhB synthesis. We emphasize that all storage compounds are of high biotechnological interest.

### The diversity of cyanobacterial central metabolism

**Figure 8:**
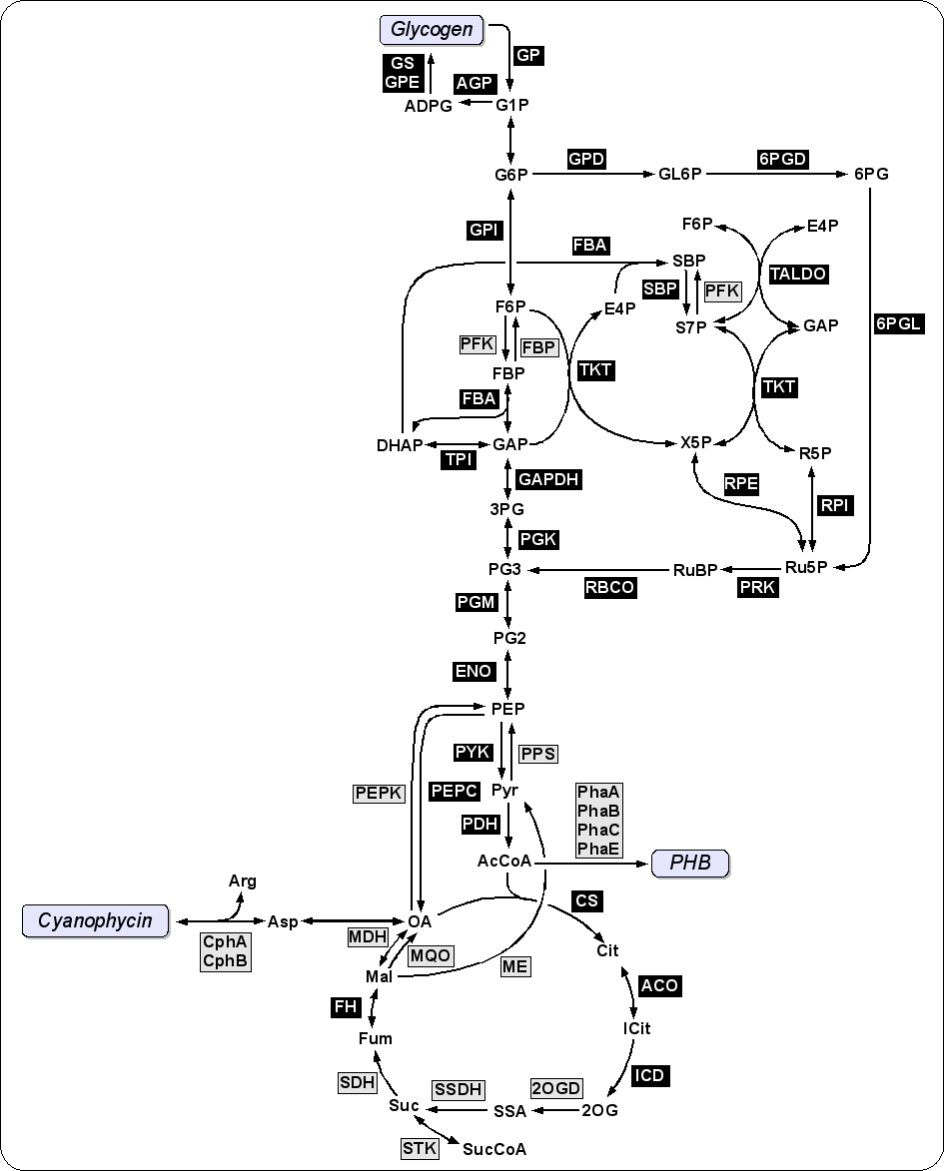
**A pathway diagram of the cyanobacterial core metabolic network**. Black boxes indicate enzymes whose corresponding CLOGs are associated with all 16 cyanobacterial strains. Grey boxes correspond to enzymes that are not annotated for one or more strains.

Beyond storage compounds, Tables 4 and 5 summarize the presence of several key enzymes within cyanobacterial central metabolism across all 16 strains considered in this study. In contrast to Table 3, the tables do not distinguish between individual CLOGs associated with the same enzymatic function. A detailed depiction of individual CLOGs is provided as supplementary material (Additional File 6). For each enzyme usually a dominant CLOG exists in addition to a smaller number of secondary CLOGs. A graphical depiction of annotated pathways is given in Figure 8. Tables 4 and 5 allow for a detailed analysis of metabolic function. First, we note that all key enzymes of the Calvin-Benson cycle, responsible for CO_2 _fixation, are annotated in all 16 strains (Table 4). Likewise, for all enzymes belonging to the pentose phosphate pathway (PPP), respective CLOGs are associated with all strains (Table 5). A more diverse picture is obtained for other key metabolic pathways. The enzyme FBP (fructose-1,6-bisphosphatase, EC 3.1.3.11) is not annotated for all strains and absent in all alpha-cyanobacteria, including the Prochlorococcus strains. However, taking into account results from a recent reconstruction and stoichiometric modeling of the strain Syn6803, the enzyme was found to be not essential for biomass formation [27]. To some extend, its function can also be substituted by the bifunctional enzyme SBP (fructose-1,6-/sedoheptulose-1,7-bisphosphatase, EC 3.1.3.37), present in all strains considered in this study. Likewise, the enzyme PFK (phosphofructokinase, EC 2.7.1.11) is not annotated for several strains, most notably again the Prochlorococcus strains. We note that PFK is essential for glycolysis, in its absence utilization of glycogen as a carbon and energy source has to proceed exclusively via the PPP. Other enzymes of the glycolytic pathway, such as FBA, GAP, PGM, and PYK are annotated for all strains (Table 4). In contrast, pyruvate metabolism, summarized in Table 5, is rather fragmented. While CLOGs annotated with the PEP carboxylase (PEPC, EC 4.1.1.31) are associated with all strains, the back reaction via the PEPKinase is rather rare and annotated only for three strains. PEPC catalyzes the anaplerotic conversion of PEP to oxaloacetate and inorganic phosphate Pi, and is essential for replenishment of TCA cycle intermediates. CLOGs annotated with the right-hand side of the TCA cycle, resulting in the formation of 2-oxoglutarate, are ubiquitous for all strains. The metabolite 2-oxoglutarate is considered to be a sensor for the nitrogen status of cyanobacteria [29] and serves as a precursor for several amino acids and nucleotides.

**Table 4:** Comparison of metabolic key enzymes: Glycolysis and Calvin Benson cycle.

	*Glycolysis*											*Calvin Benson Cycle*					
	GPI	FBP	SBP	FBA	TPI	PFK	GAPDH	PGM	ENO	PYK	PGK	RPI	TKT	TALDO	PRK	RPE	RBCO
**Aca11017**	••	••	••	••	•	•	••	• • •	•	• • •	•	•*•*	*•*	*• • •*	*•*	*•*	*••*
**Cyn51142**	*•*	*•*	*•*	*••*	*•*	*••*	*••*	*• • • • ••*	*•*	*• • •*	*•*	*•*	*•*	*• • •*	*•*	*•*	*••*
**Cyn8801**	*•*	*•*	*•*	*•*	*•*	*••*	*••*	*• • ••*	*•*	*••*	*•*	*•*	*•*	*••*	*•*	*•*	*••*
**Glo7421**	*•*	*•*	*•*	*••*	*•*	-	*••*	*• • • • •*	*•*	*• • •*	*•*	*••*	*•*	*••*	*• • •*	*•*	*••*
**Mic843**	*•*	*•*	*•*	*•*	*•*	*••*	*••*	*• • ••*	*•*	*• • •*	*•*	*•*	*• • •*	*•*	*•*	*•*	*••*
**Nos7120**	*•*	*•*	*•*	*••*	*•*	*•*	*• • •*	*• • ••*	*•*	*••*	*•*	*•*	*••*	*••*	*••*	*•*	*••*
**ProMed4**	*•*	-	*•*	*•*	*•*	-	*••*	*••*	*•*	*•*	*•*	*•*	*• • •*	*•*	*•*	*•*	*••*
**Pro9211**	*•*	-	*•*	*•*	*•*	-	*••*	*••*	*•*	*•*	*•*	*•*	*•*	*•*	*•*	*•*	*••*
**Pro9215**	*•*	-	*•*	*•*	*•*	-	*•*	*••*	*•*	*•*	*•*	*•*	*•*	*•*	*•*	*•*	*••*
**SycJA23**	*•*	*•*	*•*	*•*	*•*	*•*	*• • •*	*• • •*	*•*	*••*	*•*	*•*	*•*	*•*	*••*	*•*	*••*
**Syc7002**	*•*	*•*	*•*	*••*	*•*	*•*	*••*	*••*	*•*	*•*	*•*	*•*	*•*	*••*	*••*	*•*	*••*
**Syc7803**	*•*	-	*•*	*••*	*•*	-	*• • •*	*••*	*•*	*•*	*•*	*•*	*•*	*•*	*•*	*•*	*••*
**Syc7942**	*•*	*•*	*•*	*•*	*•*	*•*	*• • •*	*• • ••*	*•*	*•*	*•*	*•*	*•*	*•*	*•*	*•*	*••*
**Syn6803**	*•*	*•*	*•*	*••*	*•*	*••*	*••*	*• • ••*	*•*	*••*	*•*	*••*	*•*	*•*	*•*	*•*	*••*
**ThermoBP1**	*•*	*•*	*•*	*•*	*•*	*•*	*••*	*• • ••*	*•*	*••*	*•*	*•*	*•*	*•*	*•*	*•*	*••*
**Trich101**	*•*	*•*	*•*	*••*	*•*	-	*• • •*	*••*	*•*	*•*	*•*	*•*	*•*	*• • •*	*••*	*•*	*••*

A summary of the genes involved in central carbon metabolism: Glycolysis and the Calvin Benson cycle. Black dots represent the number of genes assiated with CLOGs assigned to the respective enzymatic function. The table does not distinguigh between individual CLOGs assigned to the same enzymatic function. Abbreviations are: GPI Glucose-6-phosphate isomerase (5.3.1.9), FBP Fructose-1,6-bisphosphatase (3.1.3.11), SBP Fructose-1,6-/Sedoheptulose-1,7-bisphosphatase (3.1.3.37), FBA Fructose-bisphosphate aldolase (4.1.213), TPI Triosephosphate isomerase (5.1.3.1), PFK Phosphofructokinase (2.7.1.11), GAPDH Glyceraldehyde 3-phosphate dehydrogenase (1.2.1.12/59), PGM Phosphoglycerate mutase (5.4.2.1), ENO Enolase (4.2.1.11), PYK Pyruvate kinase (2.7.1.40), PGK Phosphoglycerate kinase (2.7.2.3), RPI Ribose-5-P isomerase (5.3.1.6), TKT Transketolase (2.2.1.1), TALDO Transaldolase (2.2.1.2), PRK Phosphoribulokinase (2.7.1.19), RPE Ribulose-5-P 3-epimerase (5.1.3.1), RBCO Ribulose 1,5-bisphosphate carboxylase/oxygenase (4.1.1.39).

**Table 5:** Comparison of metabolic key enzymes: PPP, pyruvate metabolism and TCA cycle.

	*PPP*			*Pyruvate Metabolism*					*TCA Cycle*							
	GPD	6PGD	6PGL	PEPC	ME	PPS	PEPK	PDH	CS	ACO	ICD	STK	SDH	FH	MDH	MQO
**Aca11017**	*••*	••	•	•	•	• • ••	-	••	••	•	••	-	• • (•)	•	•	-
**Cyn51142**	•	•	•	•	•	• • •	•	••	•	•	•	••	• • (•)	•	•	-
**Cyn8801**	•	•	•	•	•	• • •	•	••	•	•	•	••	• • (•)	•	•	-
**Glo7421**	••	•	•	•	•	••	-	• • ••	•	•	••	-	• • •	(•)	•	-
**Mic843**	•	•	•	•	•	• • •	•	••	•	•	•	••	• • (•)	•	•	-
**Nos7120**	•	•	•	•	•	• • • • •	-	••	•	•	•	••	• • (•)	•	•	-
**ProMed4**	•	•	•	•	-	-	-	• • •	•	•	•	-	-	•	-	•
**Pro9211**	•	•	•	•	-	-	-	••	•	•	•	-	-	•	-	•
**Pro9215**	•	•	•	•	-	-	-	••	•	•	•	-	•	•	-	•
**SycJA23**	•	•	•	•	•	•	-	••	•	•	•	-	• • •	•	•	-
**Syc7002**	•	•	•	•	•	•	-	••	•	•	•	••	• • (•)	(•)	•	-
**Syc7803**	•	•	•	•	-	-	-	••	•	•	•	-	-	•	-	•
**Syc7942**	•	•	•	•	•	•	-	••	•	•	•	-	• • •	•	-	-
**Syn6803**	•	•	•	•	•	•	-	••	•	•	•	••	• • •(•)	•	•	-
**ThermoBP1**	•	•	•	•	•	••	-	••	•	•	•	-	• • •	•	-	-
**Trich101**	•	•	•	•	•	• • •	-	••	•	•	•	••	• • •	•	-	-

A summary of the genes involved in central carbon metabolism: The pentose phosphate pathway (PPP), pyruvate metabolism and the TCA cycle. Black dots represent the number of genes assiated with CLOGs assigned to the respective enzymatic function. The table does not distinguigh between individual CLOGs assigned to the same enzymatic function. Brackets indicate genes that are annotated with a different function as the respective CLOGs they are assigned to. These cases may represent erroneous annotation or bifunctional enzymes. Abbreviations are: GPD G6P dehydrogenase (1.1.1.49), 6PGD Phosphogluconate dehydrogenase (1.1.1.44), 6PGL Phosphogluconolactonase (3.1.1.31), PEPC PEP carboxylase (4.1.1.31), ME Malic enzyme (1.1.1.38), PPS Pyruvate water dikinase (2.7.9.2), PEPK PEP carboxykinase (4.1.1.49), PDH Pyruvate dehydrogenase (1.2.4.1), CS Citrate synthase (2.3.3.1), ACO Aconitase (4.2.1.3), ICD Isocitrate dehydrogenase (1.1.1.41/2), STK Succinate thiokinase (6.2.1.5), SDH Succinate dehydrogenase (1.3.99.1), FH Fumarate hydratase (4.2.1.2), MDH Malate dehydrogenase (1.1.1.37) and MQO Malate:Quinone oxidoreductase (1.1.5.4).

The subsequent steps within the TCA cycle are highly fragmented. No CLOGs are associated with the enzyme complex 2-oxogluterate dehydrogenase (OGDH), catalyzing the conversion from 2-oxogluterate to succinyl-CoA. The lack of OGDH corrosponds to the known fact that cyanobacteria do not possess a conventional TCA cycle. Nonetheless, it is generally believed that the TCA cycle is able to carry cyclic flux and is therefore able to fulfill its respiratory function in the absence of light. Within the reconstruction of Knoop *et al*. [27] the cyclic flux through the TCA cycle was realized by a metabolic bypass that complements the missing enzyme complex, via three steps involving glutamate decarboxylase (EC 4.1.1.15), 4-aminobutyrate transaminase (EC 2.6.1.19) and succinate-semialdehyde dehydrogenase (SSDH, EC 1.2.1.16). However, only recently, a proper bypass of the OGDH was identified. Zhang and Bryant report that in the strain Syc7002 a novel 2-oxoglutarate decarboxylase (2OGD, EC 4.1.1.71) and succinate-semialdehyde dehydrogenase (SSDH, EC 1.2.1.16) together convert 2-oxogluterate to succinate and thus close the TCA cycle (Zhang and Bryant, 2011)[30]. The respective CLOGs are shared between all strains, except the three Prochlorococus strains, Syc7942, and Syc7803, see Table 5 for an overview. The existence of a bypass also explains that the succinate thiokinase (STK, EC 6.2.1.5), otherwise an essential enzyme within the TCA cycle, is only annotated for a small number of strains. We speculate that in these few cases the enzyme STK serves mainly to produce succinyl-CoA. Furthermore, several strains lack CLOGs annotated with the enzyme succinate dehydrogenase (SDH, EC 1.3.99.1). For these cyanobacteria cellular respiration via the TCA cycle is significally impaired. CLOGs associated with the enzyme fumarate hydratase (FH, EC 4.2.1.2) are present for all cyanobacteria. However, for Syc7002 and Glo7421, the original genes are annotated differently, representing either erroneous annotation or a bifunctional enzyme. Extrapolating results from a metabolic model of Syn6803 (Knoop *et al*., 2010), the enzyme FH is essential to recycle fumarate, which is an obligate byproduct during growth. In addition, several other enzymes are only annotated in a subset of the considered strains. In particular, the malic enzyme (ME, EC 1.1.1.38) and the malate dehydrogenase (MDH, EC 1.1.1.37) are absent in all alpha-cyanobacteria. However, for these strains, the function of the latter can be substituted by the malate:quinone oxidoreductase (MQH, EC 1.1.5.4), which catalyzes the irreversible oxidation of malate to oxaloacetate. Overall, the assigmment of CLOGs to the 16 cyanobacterial strains considered here reveals a complex picture and considerable metabolic diversity in enzymes of the core carbon metabolism.

## Conclusions

The rapidly increasing number of complete microbial genomes offers new possibilities to understand microbial diversity in complex environments. In this work, we have presented a whole genome analysis of multiple phototrophic cyanobacteria, with the aim to gain insight into the diversity of cyanobacterial metabolism from a genome perspective. Cyanobacteria exhibit an enormous metabolic diversity and occur in almost all environments where light is available, and are therefore particularly suited for a comparative analysis of genetic diversity. The basis of our analysis was the definition of clusters of likely ortholog genes (CLOGs), and how these are distributed across the 16 cyanobacterial strains considered in this study. We found that of the 21238 distinct CLOGs identified across all cyanobacterial strains, the majority (approximately 65%) consist of single genes that have no likely ortholog in any other considered strain. About 3% of CLOGs are assigned to all strains, constituting the core genome shared among all strains considered in this study. The remaining CLOGs are assigned to more than one, but not all strains. We note that the set of core CLOGs does not represent a minimal set of genes to sustain Life.

Several conclusions can be drawn from the distribution of CLOGs: First, we find no indication that the pan-genome of cyanobacteria is closed. Rather, the number of total CLOGs increases indicating that no small number of sequenced strains is sufficient to explore the full diversity of the cyanobacterial genome. While such extrapolations must be taken with caution [17], these result at least provide a strong incentive for further sequencing projects. Second, the set of core and unique CLOGs differs in with respect to enriched annotations. Core CLOGs exhibit a significant enrichment of genes with annotations that are commonly associated with household functions, such as "translation", "DNA repair", "gene expression", "RNA processing/modification", diverse transporting processes, as well as several metabolic and biosynthetic processes. The annotation of genes of unique CLOGs are enriched with terms pointing to more specific functions, such as various regulatory processes. Furthermore, we found that codon frequencies are different for core CLOGs between different strains, indicating adaptation of codon usage to the respective organisms.

A focus of our work was to explore the metabolic diversity of cyanobacteria from a genomic perspective. CLOGs assigned to metabolic functions are strongly overrepresented within the set of core CLOGs common to all strains considered in this study. Investigating the distribution of individual CLOGs in more detail, our analysis revealed a diverse picture of the presence of core metabolic pathways within all trains. Several key pathways in central metabolism are highly conserved, such as the pentose phosphate pathway and the Calvin-Benson cycle. However, other parts of the metabolic network, most notably pyruvate metabolism and the TCA cycle, are highly fragmented. In contrast to conventional functional annotation, the annotation of enzymatic function offers the advantage that the functional context of assigned CLOGs, in terms of pathways and adjacent reactions, can be taken into account. Therefore the analysis allows to assess the presence or absence of certain enzymes in terms of metabolic function, providing stronger criteria to judge erroneous annotation or reliability of the associated CLOGs. For example, the incompletely annotated TCA cycle of cyanobacteria puts constraints on its ability to provide the precursors for cellular respiration. This deficiency, in term, has consequences for the functional role of other assigned reactions, such as the succinate thiokinase (STK). The latter is therefore unlikly to assume its usual role, and correspondingly is annotated only for a small number of strains. In this respect, we consider our analysis also as a first step in automated network reconstruction. Large-scale models of cellular metabolism are becoming increasingly important for a variety of biotechnological applications, but are currently often restricted to a small number of model strains [27,31]. Reconstruction of multiple strains can greatly benefit from a thorough analysis of metabolic diversity found among the set of already sequenced cyanobacteria. We expect that an iterative process, from genome analysis to model construction will eventually lead to a leap in understanding of the metabolic and ecological capabilities of bacterial species and to advanced eco-systems biology.

## Methods

### Genome sequences of cyanobacterial strains

For this study, the following 16 strains were selected: *Acaryochloris marina *MBIC11017 (Aca11017); *Cyanothece ATCC 51142 *(Cyn51142); *Cyanothece *PCC 8801 (Cyn8801); *Gloeobacter violaceus *PCC 7421 (Glo7421); *Microcystis aeruginosa *NIES-843 (Mi843); *Nostoc sp*. PCC 7120 (Nos7120); *Prochlorococcus marinus *MED4 (ProMED4); *Prochlorococcus marinus *MIT 9211 (Pro9211); *Prochlorococcus marinus *MIT 9215 (Pro9215); *Synechococcus JA-2-3B_a *(SycJA23); *Synechococcus sp*. PCC 7002 (Syc7002); *Synechococcus sp*. WH7803 (Syc7803); *Synechococcus elongatus *PCC 7942 (Syc7942); *Synechocystis sp*. PPC 6803 (Syn6803); *Thermosynechococcus elongatus *BP-1 (ThermoBP1); *Trichodesmium erythraeum *IMS101 (Trich101). The respective chromosomal genomes were extracted from GenBank in August 2010 (http://www.ncbi.nlm.nih.gov/genbank) [32]. Plasmids of the strains were not considered. The decision to neglect these small additional DNA fragments was taken since there is insufficient knowledge about the genes encoded on them and to avoid possible bias favoring genomes with plasmids enriched of genes possibly gained by horizontal gene transfer compared to strains without one [33]. Our choice of strains was based on the results described by Gupta and Mathews (2010). Our aim was to cover cyanobacterial diversity, as presented as a maximum-likelihood distance tree for sequenced cyanobacteria based on concatenated sequences for 44 conserved protein. The tree is reproduced as Additional File 7 highlighting the position of the 16 selected strains within the tree. An overview of the properties of the selected strains is given in Table 1.

### Definition of clusters of likely ortholog genes (CLOGs)

To identify ortholog genes, we performed an all-against-all comparison of all 16 cyanobacterial strains. For each possible combination of species A and B ortholog genes are identified using a method similar to the KEGG Automatic Annotation Server [34]. First all genes of A are compared to each gene in B and vice versa using blastp. Hits with a bit score below 50 bits are rejected. The bidirectional hit rate (BHR) for a gene pair *a *and *b *is computed as

$$BHR=\left(\frac{S_{a,b}}{S_{b}^{bestA}}\right)\times \left(\frac{S_{b,a}}{S_{a}^{bestB}}\right)$$

where *S*_*a*,*b *_is the blastp score of *a *versus *b *and $S_{b}^{bestA}$ is the best score of *b *against any gene in A (which may be different to *a*). The value of BHR is unity for genes which are mutually best hits in both directions, and lower otherwise. The set of genes for which the BHR is calculated includes genes located on the same genome. To favor cross-genome orthologs in the further steps of our analysis, the BHR of genes located on the same genome is artificially restricted to values up to 0.95, even when the actual value is higher. All gene pairs with a BHR above or equal 0.95 are classified as putative orthologs.

In a second step, the gene pairs are then clustered by merging all genes which are identified as putative orthologs. To avoid clusters where two genes have a low BHR but are weakly connected through a third gene, all genes in a pre-cluster are clustered again with UPGMA (Unweighted Pair Group Method with Arithmetic mean) and a minimal BHR of 0.75 [35]. This is accomplished by clustering the closest entities - the one with the highest BHR - and recalculating the BHR to all other entitiesCusing the following equation

$$BHR_{X,C}=\frac{|A|\times BHR_{A,C}+|B|\times BHR_{B,C}}{|A|+|B|},$$

where *X *is the new entity merged of entities *A *and *B*, |*A*| is the size of *A*, and *BHR*_*X*,*C *_is the BHR of two entities, until all pairs of entities have a BHR below 0.75. Using our procedure, each gene is assigned to a single cluster.

### Analysis of codon usage

The codon usage of a group g of genes was calculated by randomly selecting 100 different genes within this group, such that each gene has a size of at least 50 amino acids. For each encoding triplet *t *we calculated the relative frequency *f*_*t*,*g *_with which the triplet codes for its corresponding amino acid across all selected genes *g*. Stop codons were not taken into account. The differences in codon usage of two groups *i *and *j *where calculated according to

$$d_{i,j}^{\prime 2}=\overset{n}{\underset{t=all\;coding\;triplets}{\sum }}{\left(f_{t,i}-f_{t,j}\right)}^{2}.$$

To account for selection bias, randomized gene selection within the groups and calculation of *d'*^2 ^was 100 P repeated 100 times and averaged $d_{i,j}^{2}=\frac{1}{100}\overset{100}{\underset{1}{\sum }}d_{i,j}^{\prime 2}$. To quantify the difference in codon usage between the set of of core and unique genes, we use the ratio *r_x _*of the sum of squared differences in codon usage between core/core and core/unique genes for each strain,

$$r_{x}=\frac{d_{core\left(x\right),unique\left(x\right)}^{2}}{d_{core\left(x\right),core\left(x\right)}^{2}}$$

The values are provided in Additional File 4. To verify if the differences in codon usage between core and unique genes within one species are statistically significant we used a two-sample Kolmogorov-Smirnov test with 100 repeats of $d_{core\left(x\right),core\left(x\right)}^{2}$ and $d_{core\left(x\right),unique\left(x\right)}^{2}$. The test rejected the hypothesis that both samples came from the same distribution for all strains with an asymptotic p-value of *p <*4*e*^-27 ^for Pro9215 and *p <*4*e*^-43 ^for all other strains.

### Enrichment of GO annotation

To investigate functional differences between core and uniqu CLOGs, each CLOG was assigned to the functional annotation provided by the Gene Onthology (GO) database [23,36] (effective January 2011) for every constituent gene within the respective CLOG. For enrichment analysis of GO terms the TopGO software was used [37], which is available as part of the Bioconductor R packages (http://www.bioconductor.org). Here we chose the parent-child algorithm [38] with standard parameters and Fisher's exact test. For the calculation of p-values only CLOGs with assigned GO term where taken into account. The complete list of all enriched terms with p-values *< e*^-4 ^for core and unique CLOGs is provided as Additional File 3.

### Phylogenetic tree analysis

The trees presented in this work were created with PHYLIP (PHYLogeny Inference Package version 3.69) by Felsenstein [24]. For the 16S-RNA comparison (Figure 4A), a tree in newick format was constructed with the Ribosomal Database Project web site (http://rdp.cme.msu.edu/) [39]. The tree was then plotted with DRAWGRAM of PHYLIP.

For the tree based on shared clusters (Figure 4B), we built a similarity matrix for all strains, where the similarity between two strains is calculated by the number of the shared cluster divided by the number of clusters where at least one of the two strains participate. We did not take into account core clusters (which are the same for all strains) and unique clusters. The latter also serves to minimize size bias. From this matrix we calculated a distance matrix by substracting each entry from the maximal entry. The distance matrix was then converted into a tree using NEIGHBOR of PHYLIP and subsequently plottet again with DRAWGRAM. In both trees, Aca11017 was used as outgroup.

### Assigments of Metabolic Function

Metabolic functions were assigned to the CLOGS by matching the occurring genes to the KEGG database (release date 19. october 2010) [40]. CLOGs with at least one gene associated to an enzymatic function in the KEGG database, are labeled with the respective EC number. In case that a CLOG contains genes which are assigned to different EC numbers we annotate this CLOG with multiple EC numbers, unless one of the numbers is just an incomplete form of the other (i.e. 3.7.-.- and 3.7.4.21). Consequentially the total number of distinct EC numbers does not exactly correspond to the number of metabolic CLOGS. For enzyme complexes consisting of multiple subunits, which are encoded by several genes and are therefore associated with different CLOGs, the EC number of the corresponding enzyme is assigned to each of the CLOGs. We note that EC numbers do not strictly correspond to genuine small-molecule metabolic function, since the EC nomenclature also includes general enzymatic activity, such as protein kinases and RNA or DNA polymerases.

## Authors' contributions

CB, HK, IMA and RS participated in the design of the study. CB and HK jointly carried out genome comparisons and bioinformatics analysis. HK perfomed metabolic assigments. RS participated in the coordination of the study and helped to draft the manuscript. All authors read and approved the final manuscript.

## Supplementary Material

Additional file 1 **Text file of CLOGs**. Tab separated text file containing all CLOGs found by our methodes. Each line represents one CLOG, the entries for each strain are separated by tabs. Genes are annotated with the respective EC number, is applicable. If one strain has more than one entry in a CLOG, the genes are separated by a tilde. The last column summarizes all EC numbers denoted to genes in this CLOG and the frequency of appearance. Multiple ECs in one CLOG are separated by a hash. Click here for file

Additional file 2 **Table of strains assigned to CLOGs of different sizes**. The table provides the number of CLOGs assigned to each strain. The fraction of CLOGs associated with one or more EC number is given in brackets. The size of a CLOG is determined by the number of strains that it is associated with. Click here for file

Additional file 3 **Table of enriched Gene Onthology terms**. An excel file which contains the list of enriched GO terms for genes of the core CLOGs as well as for genes of the unique CLOGs. For each enriched term the table gives: the GO specific ID; the term in clear text; the total number of CLOGs annotated with the term across all clusters; the number of CLOGs in the particular set annotated with the term; the expected number of CLOGs in the current set annotated with the term, given an uniform distribution; the significance level of the enrichment, calculated with fisher's exact test. Only results with a p-value below 1e-3 are shown. Each list is divided into the three GO domains "biological process", "cellular component", and "molecular function". Click here for file

Additional file 4 **Table of differential codon usage**. The table shows the differences in codon usage of core and unique genes across all 16 cyanobacterial strains. Each number indicates the difference in codon usage of the core genes of one strain (row) compared to the core or unique genes of one strain (columns) and is calculated as described in Methods. Click here for file

Additional file 5 **Differences in EC annotation**. The pdf contains a list of EC numbers corresponding to groups A and D in Figure 7, respectively. Click here for file

Additional file 6 **Tables of CLOGs related to key enzymes of central metabolism**. The table provides the CLOGs associated to enzymes involved in the storage metabolism, glycolysis, Calvin Benson cycle, PPP, pyruvate metabolism and TCA cycle. For each strain a dot is representing a strain specific gene which can be found in the CLOG with the corresponding enzymatic function. Bracketed dots represent genes, which are assigned to that CLOG, but differ in annotation and most likely in function. Click here for file

Additional file 7 **Annotated phylogenetic tree**. Shown is a maximum-likelihood distance tree for sequenced cyanobacteria reproduced from Gupta et al. 2010 [9]. Strains chosen for analysis are indicated by red arrows. Click here for file

## References

[B1] Ducat DC Way JC Silver PA Engineering cyanobacteria to generate high-value products Trends Biotechnol 2011 29 2 95 103 10.1016/j.tibtech.2010.12.003 21211860

[B2] Hess WR Genome analysis of marine photosynthetic microbes and their global role Curr Opin Biotechnol 2004 15 3 191 8 10.1016/j.copbio.2004.03.007 15193326

[B3] Hess WR Cyanobacterial genomics for ecology and biotechnology Curr Opin Microbiol 2011 14 5 608 14 10.1016/j.mib.2011.07.024 21840247

[B4] Mulkidjanian AY Koonin EV Makarova KS Mekhedov SL Sorokin A Wolf YI Dufresne A Partensky F Burd H Kaznadzey D Haselkorn R Galperin MY The cyanobacterial genome core and the origin of photosynthesis Proc Natl Acad Sci USA 2006 103 35 13126 31 10.1073/pnas.0605709103 16924101 PMC1551899

[B5] Raymond J Zhaxybayeva O Gogarten JP Gerdes SY Blankenship RE Whole-genome analysis of photosynthetic prokaryotes Science 2002 298 5598 1616 20 10.1126/science.1075558 12446909

[B6] Zhaxybayeva O Gogarten JP Charlebois RL Doolittle WF Papke RT Phylogenetic analyses of cyanobacterial genomes: quantification of horizontal gene transfer events Genome Res 2006 16 9 1099 108 10.1101/gr.5322306 16899658 PMC1557764

[B7] Shi T Falkowski PG Genome evolution in cyanobacteria: the stable core and the variable shell Proc Natl Acad Sci USA 2008 105 7 2510 5 10.1073/pnas.0711165105 18268351 PMC2268167

[B8] Gupta RS Pereira M Chandrasekera C Johari V Molecular signatures in protein sequences that are characteristic of cyanobacteria and plastid homologues Int J Syst Evol Microbiol 2003 53 Pt 6 1833 42 14657112 10.1099/ijs.0.02720-0

[B9] Gupta RS Mathews DW Signature proteins for the major clades of Cyanobacteria BMC Evol Biol 2010 10 24 10.1186/1471-2148-10-24 20100331 PMC2823733

[B10] Venter JC Remington K Heidelberg JF Halpern AL Rusch D Eisen JA Wu D Paulsen I Nelson KE Nelson W Fouts DE Levy S Knap AH Lomas MW Nealson K White O Peterson J Hoffman J Parsons R Baden-Tillson H Pfannkoch C Rogers YH Smith HO Environmental genome shotgun sequencing of the Sargasso Sea Science 2004 304 5667 66 74 10.1126/science.1093857 15001713

[B11] Rusch DB Martiny AC Dupont CL Halpern AL Venter JC Characterization of Prochlorococcus clades from iron-depleted oceanic regions Proc Natl Acad Sci USA 2010 107 37 16184 9 10.1073/pnas.1009513107 20733077 PMC2941326

[B12] Tatusov RL Koonin EV Lipman DJ A genomic perspective on protein families Science 1997 278 5338 631 7 10.1126/science.278.5338.631 9381173

[B13] Coleman ML Sullivan MB Martiny AC Steglich C Barry K Delong EF Chisholm SW Genomic islands and the ecology and evolution of Prochlorococcus Science 2006 311 5768 1768 70 10.1126/science.1122050 16556843

[B14] Hogg JS Hu FZ Janto B Boissy R Hayes J Keefe R Post JC Ehrlich GD Characterization and modeling of the Haemophilus influenzae core and supragenomes based on the complete genomic sequences of Rd and 12 clinical nontypeable strains Genome Biol 2007 8 6 R103 10.1186/gb-2007-8-6-r103 17550610 PMC2394751

[B15] Baumdicker F Hess W Pfaffelhuber P The diversity of a distributed genome in bacterial populations The Annals of Applied Probability 2010 20 1567 1606 10.1214/09-AAP657

[B16] Larsson J Nylander JA Bergman B Genome fluctuations in cyanobacteria reflect evolutionary, developmental and adaptive traits BMC Evol Biol 2011 11 187 10.1186/1471-2148-11-187 21718514 PMC3141437

[B17] Kislyuk AO Haegeman B Bergman NH Weitz JS Genomic fluidity: an integrative view of gene diversity within microbial populations BMC Genomics 2011 12 32 10.1186/1471-2164-12-32 21232151 PMC3030549

[B18] Tatusov RL Natale DA Garkavtsev IV Tatusova TA Shankavaram UT Rao BS Kiryutin B Galperin MY Fedorova ND Koonin EV The COG database: new developments in phylogenetic classification of proteins from complete genomes Nucleic Acids Res 2001 29 22 8 10.1093/nar/29.1.22 11125040 PMC29819

[B19] Lapierre P Gogarten JP Estimating the size of the bacterial pan-genome Trends Genet 2009 25 3 107 10 10.1016/j.tig.2008.12.004 19168257

[B20] Tettelin H Riley D Cattuto C Medini D Comparative genomics: the bacterial pan-genome Curr Opin Microbiol 2008 11 5 472 7 10.1016/j.mib.2008.09.006 19086349

[B21] Tettelin H Masignani V Cieslewicz MJ Donati C Medini D Ward NL Angiuoli SV Crabtree J Jones AL Durkin AS Deboy RT Davidsen TM Mora M Scarselli M Margarit y Ros I Peterson JD Hauser CR Sundaram JP Nelson WC Madupu R Brinkac LM Dodson RJ Rosovitz MJ Sullivan SA Daugherty SC Haft DH Selengut J Gwinn ML Zhou L Zafar N Khouri H Radune D Dimitrov G Watkins K O'Connor KJ Smith S Utterback TR White O Rubens CE Grandi G Madoff LC Kasper DL Telford JL Wessels MR Rappuoli R Fraser CM Genome analysis of multiple pathogenic isolates of Streptococcus agalactiae: implications for the microbial "pan-genome" Proc Natl Acad Sci USA 2005 102 39 13950 5 10.1073/pnas.0506758102 16172379 PMC1216834

[B22] Mira A Martin-Cuadrado AB D'Auria G Rodriguez-Valera F The bacterial pan-genome:a new paradigm in microbiology Int Microbiol 2010 13 2 45 57 20890839 10.2436/20.1501.01.110

[B23] Ashburner M Ball CA Blake JA Botstein D Butler H Cherry JM Davis AP Dolinski K Dwight SS Eppig JT Harris MA Hill DP Issel-Tarver L Kasarskis A Lewis S Matese JC Richardson JE Ringwald M Rubin GM Sherlock G Gene ontology: tool for the unification of biology. The Gene Ontology Consortium Nat Genet 2000 25 25 9 10.1038/75556 10802651 PMC3037419

[B24] Felsenstein J PHYLIP (Phylogeny Inference Package) version 3.6 2004

[B25] Johnson Z Lin Y Prochlorococcus: Approved for export Proceedings of the National Academy of Sciences 2009 106 26 10400 10401 10.1073/pnas.0905187106 PMC2705537 19553202

[B26] Martiny A Kathuria S Berube P Widespread metabolic potential for nitrite and nitrate assimilation among Prochlorococcus ecotypes Proceedings of the National Academy of Sciences 2009 106 26 10787 10792 10.1073/pnas.0902532106 PMC2705535 19549842

[B27] Knoop H Zilliges Y Lockau W Steuer R The metabolic network of Synechocystis sp. PCC 6803: systemic properties of autotrophic growth Plant Physiol 2010 154 410 22 10.1104/pp.110.157198 20616194 PMC2938163

[B28] Miyake M Takase K Narato M Khatipov E Schnackenberg J Shirai M Kurane R Asada Y Polyhydroxybutyrate production from carbon dioxide by cyanobacteria Appl Biochem Biotechnol 2000 84-86 991 1002 10.1385/ABAB:84-86:1-9:991 10849853

[B29] Muro-Pastor MI Reyes JC Florencio FJ Ammonium assimilation in cyanobacteria Photosynth Res 2005 83 2 135 50 10.1007/s11120-004-2082-7 16143848

[B30] Zhang S Bryant DA The tricarboxylic acid cycle in cyanobacteria Science 2011 334 6062 1551 3 PMID: 22174252 10.1126/science.1210858 22174252

[B31] Oberhardt MA Palsson BO Papin JA Applications of genome-scale metabolic reconstructions Mol Syst Biol 2009 5 320 19888215 10.1038/msb.2009.77 PMC2795471

[B32] Benson DA Karsch-Mizrachi I Lipman DJ Ostell J Wheeler DL GenBank Nucleic Acids Res 2008 36 Database D25 30 18073190 10.1093/nar/gkm929 PMC2238942

[B33] Kaneko T Nakamura Y Sasamoto S Watanabe A Kohara M Matsumoto M Shimpo S Yamada M Tabata S Structural analysis of four large plasmids harboring in a unicellular cyanobacterium, Synechocystis sp. PCC 6803 DNA Res 2003 10 5 221 8 10.1093/dnares/10.5.221 14686584

[B34] Moriya Y Itoh M Okuda S Yoshizawa AC Kanehisa M KAAS: an automatic genome annotation and pathway reconstruction server Nucleic Acids Res 2007 35 Web Server W182 5 17526522 10.1093/nar/gkm321 PMC1933193

[B35] Sokal R Michener C A statistical method for evaluating systematic relationships University of Kansas Science Bulletin 1958 38 1409 38

[B36] Harris MA Clark J Ireland A Lomax J Ashburner M Foulger R Eilbeck K Lewis S Marshall B Mungall C Richter J Rubin GM Blake JA Bult C Dolan M Drabkin H Eppig JT Hill DP Ni L Ringwald M Balakrishnan R Cherry JM Christie KR Costanzo MC Dwight SS Engel S Fisk DG Hirschman JE Hong EL Nash RS Sethuraman A Theesfeld CL Botstein D Dolinski K Feierbach B Berardini T Mundodi S Rhee SY Apweiler R Barrell D Camon E Dimmer E Lee V Chisholm R Gaudet P Kibbe W Kishore R Schwarz EM Sternberg P Gwinn M Hannick L Wortman J Berriman M Wood V de la Cruz N Tonellato P Jaiswal P Seigfried T White R The Gene Ontology (GO) database and informatics resource Nucleic Acids Res 2004 32 Database D258 61 14681407 10.1093/nar/gkh036 PMC308770

[B37] Alexa A Rahnenfuhrer J Lengauer T Improved scoring of functional groups from gene expression data by decorrelating GO graph structure Bioinformatics 2006 22 13 1600 7 10.1093/bioinformatics/btl140 16606683

[B38] Grossmann S Bauer S Robinson PN Vingron M Improved detection of overrepresentation of Gene-Ontology annotations with parent child analysis Bioinformatics 2007 23 22 3024 31 10.1093/bioinformatics/btm440 17848398

[B39] Cole JR Wang Q Cardenas E Fish J Chai B Farris RJ Kulam-Syed-Mohideen AS McGarrell DM Marsh T Garrity GM Tiedje JM The Ribosomal Database Project: improved alignments and new tools for rRNA analysis Nucleic Acids Res 2009 37 Database D141 5 19004872 10.1093/nar/gkn879 PMC2686447

[B40] Ogata H Goto S Sato K Fujibuchi W Bono H Kanehisa M KEGG: Kyoto Encyclopedia of Genes and Genomes Nucleic Acids Res 1999 27 29 34 10.1093/nar/27.1.29 9847135 PMC148090

[B41] Badger M Hanson D Price G Evolution and diversity of CO2 concentrating mechanisms in cyanobacteria Functional Plant Biology 2002 29 407 16 10.1071/PP01210 32689463

[B42] Markowitz VM Chen IM Palaniappan K Chu K Szeto E Grechkin Y Ratner A Anderson I Lykidis A Mavromatis K Ivanova NN Kyrpides NC The integrated microbial genomes system: an expanding comparative analysis resource Nucleic Acids Res 2010 38 Database D382 90 19864254 10.1093/nar/gkp887 PMC2808961

[B43] Rippka R Deruelles J Waterbury J Herdman M Stanier R Generic assignments, strain histories and properties of pure cultures of cyanobacteria Journal of General Microbiology 1979 111 1

