# Supplementary material for: The diversity of cyanobacterial metabolism: genome analysis of multiple phototrophic microorganisms
Source: BMC Genomics. 2012 Feb 2;13:56. doi: 10.1186/1471-2164-13-56 (PMC3369817; doi:10.1186/1471-2164-13-56)
Supplement: Additional file 2 — Table of strains assigned to CLOGs of different sizes. The table provides the number of CLOGs assigned to each strain. The fraction of CLOGs associated with one or more EC number is given in brackets. The size of a CLOG is determined by the number of strains that it is associated with. [file 1471-2164-13-56-S2.DOC]

|  | **Number of strains associate to CLOGs** | | | | | | | | | | | | | | | |
| --- | --- | --- | --- | --- | --- | --- | --- | --- | --- | --- | --- | --- | --- | --- | --- | --- |
| **Strains** | **1** | **2** | **3** | **4** | **5** | **6** | **7** | **8** | **9** | **10** | **11** | **12** | **13** | **14** | **15** | **16** |
| Aca11017 | 2458 (69) | 639 (67) | 260 (36) | 195 (35) | 167 (29) | 141 (35) | 115 (34) | 108 (27) | 117 (32) | 131 (43) | 145 (49) | 156 (44) | 93 (28) | 87 (30) | 120 (55) | 660 (364) |
| Mic843 | 2029 (21) | 611 (41) | 258 (28) | 202 (23) | 178 (23) | 138 (23) | 149 (38) | 120 (21) | 129 (31) | 152 (51) | 142 (44) | 164 (44) | 96 (28) | 90 (32) | 129 (59) | 660 (364) |
| Nos7120 | 1310 (45) | 733 (78) | 328 (35) | 247 (41) | 235 (34) | 180 (39) | 171 (48) | 139 (32) | 138 (33) | 152 (49) | 149 (49) | 168 (46) | 96 (27) | 91 (31) | 123 (56) | 660 (364) |
| Cyn51142 | 953 (28) | 593 (37) | 317 (32) | 266 (34) | 259 (41) | 180 (34) | 183 (51) | 140 (34) | 141 (32) | 150 (44) | 147 (47) | 163 (42) | 98 (28) | 89 (30) | 126 (57) | 660 (364) |
| Cyn8801 | 630 (24) | 591 (39) | 307 (28) | 252 (34) | 227 (28) | 168 (32) | 163 (44) | 136 (25) | 137 (35) | 150 (48) | 144 (45) | 165 (46) | 99 (29) | 90 (32) | 129 (59) | 660 (364) |
| Glo7421 | 1606 (79) | 504 (60) | 195 (34) | 124 (27) | 100 (20) | 96 (21) | 74 (21) | 65 (14) | 86 (28) | 85 (32) | 79 (30) | 133 (41) | 78 (27) | 59 (17) | 88 (43) | 660 (364) |
| Trich101 | 1256 (37) | 436 (42) | 181 (24) | 156 (24) | 161 (24) | 122 (21) | 119 (27) | 87 (22) | 109 (28) | 131 (46) | 132 (41) | 156 (42) | 93 (25) | 85 (29) | 120 (53) | 660 (364) |
| Syn6803 | 406 (20) | 292 (35) | 138 (18) | 168 (24) | 127 (19) | 114 (26) | 135 (39) | 119 (29) | 123 (30) | 141 (46) | 137 (42) | 162 (45) | 92 (24) | 88 (32) | 122 (58) | 660 (364) |
| Syc7002 | 356 (3) | 210 (21) | 106 (9) | 112 (15) | 117 (15) | 115 (25) | 135 (34) | 109 (28) | 122 (34) | 143 (46) | 131 (37) | 161 (42) | 92 (25) | 87 (32) | 115 (50) | 660 (364) |
| SycJA23 | 740 (27) | 250 (35) | 67 (21) | 75 (17) | 66 (11) | 55 (10) | 67 (12) | 56 (16) | 72 (19) | 100 (28) | 113 (37) | 152 (40) | 77 (23) | 65 (18) | 110 (50) | 660 (364) |
| Syc7942 | 436 (16) | 173 (22) | 90 (21) | 85 (18) | 78 (19) | 77 (17) | 79 (22) | 70 (16) | 87 (22) | 113 (34) | 131 (40) | 168 (44) | 95 (26) | 92 (33) | 127 (58) | 660 (364) |
| Syc7803 | 660 (31) | 166 (24) | 77 (14) | 201 (39) | 56 (20) | 50 (15) | 34 (12) | 38 (14) | 36 (14) | 36 (10) | 50 (22) | 87 (21) | 96 (26) | 92 (33) | 128 (59) | 660 (364) |
| ThermoBP1 | 356 (6) | 171 (16) | 78 (13) | 69 (19) | 61 (8) | 58 (13) | 64 (11) | 65 (19) | 87 (19) | 111 (35) | 122 (38) | 149 (42) | 87 (26) | 76 (30) | 126 (58) | 660 (364) |
| Pro9215 | 236 (11) | 318 (22) | 110 (8) | 186 (40) | 33 (11) | 32 (10) | 25 (13) | 21 (8) | 20 (10) | 21 (9) | 28 (17) | 34 (8) | 36 (10) | 65 (25) | 126 (58) | 660 (364) |
| ProMED4 | 194 (6) | 310 (21) | 109 (4) | 189 (39) | 36 (12) | 35 (11) | 23 (11) | 20 (8) | 18 (10) | 23 (11) | 29 (17) | 40 (6) | 39 (11) | 71 (26) | 126 (57) | 660 (364) |
| Pro9211 | 284 (9) | 131 (16) | 103 (5) | 185 (39) | 34 (11) | 29 (10) | 18 (10) | 19 (7) | 18 (10) | 21 (8) | 26 (17) | 42 (11) | 46 (14) | 89 (32) | 120 (55) | 660 (364) |

Table S2: The table provides the number of CLOGs assigned to each strain. The fraction of CLOGs associated with one or more EC number is given in brackets. The size of a CLOG is determined by the number of strains that it is associated with.
