# Supplementary material for: The diversity of cyanobacterial metabolism: genome analysis of multiple phototrophic microorganisms
Source: BMC Genomics. 2012 Feb 2;13:56. doi: 10.1186/1471-2164-13-56 (PMC3369817; doi:10.1186/1471-2164-13-56)
Supplement: Additional file 4 — Table of differential codon usage. The table shows the differences in codon usage of core and unique genes across all 16 cyanobacterial strains. Each number indicates the difference in codon usage of the core genes of one strain (row) compared to the core or unique genes of one strain (columns) and is calculated as described in Methods. [file 1471-2164-13-56-S4.DOC]

|  | **Aca11017** | | **Cyn51142** | | **Cyn8801** | | **Glo7421** | | **Mic843** | | **Nos7120** | | **ProMED4** | | **Pro9211** | | **Pro9215** | | **SycJA23** | | **Syc7002** | | **Syc7803** | | **Syc7942** | | **Syn6803** | | **ThermoBP1** | | **Trich101** | |
| --- | --- | --- | --- | --- | --- | --- | --- | --- | --- | --- | --- | --- | --- | --- | --- | --- | --- | --- | --- | --- | --- | --- | --- | --- | --- | --- | --- | --- | --- | --- | --- | --- |
|  | **core** | **unique** | **core** | **unique** | **core** | **unique** | **core** | **unique** | **core** | **unique** | **core** | **unique** | **core** | **unique** | **core** | **unique** | **core** | **unique** | **core** | **unique** | **core** | **unique** | **core** | **unique** | **core** | **unique** | **core** | **unique** | **core** | **unique** | **core** | **unique** |
| **Aca11017 (core)** | 0.017 | 0.1116 | 0.8841 | 1.2427 | 0.6407 | 1.0652 | 3.6624 | 2.4008 | 0.4882 | 0.566 | 0.5685 | 0.7564 | 2.5298 | 2.3987 | 1.4077 | 1.5409 | 2.4038 | 2.4294 | 1.8118 | 1.1799 | 0.4109 | 0.1969 | 2.4297 | 1.2328 | 0.9785 | 0.4502 | 0.37 | 0.2747 | 0.4443 | 0.3509 | 1.4577 | 1.4291 |
| **Cyn51142 (core)** | 0.8695 | 0.7285 | 0.0167 | 0.2278 | 0.1004 | 0.2469 | 6.813 | 5.0739 | 0.4773 | 0.3534 | 0.6131 | 0.4578 | 1.3623 | 1.3826 | 0.9879 | 1.1517 | 1.3347 | 1.3802 | 4.1901 | 3.2151 | 1.218 | 0.6724 | 5.1758 | 3.486 | 2.8748 | 1.9409 | 1.0639 | 0.4627 | 1.7762 | 1.7805 | 0.6812 | 0.6812 |
| **Cyn8801 (core)** | 0.641 | 0.5643 | 0.1 | 0.3957 | 0.0189 | 0.3418 | 5.9938 | 4.4485 | 0.306 | 0.3333 | 0.5659 | 0.534 | 1.6841 | 1.6757 | 1.1598 | 1.3324 | 1.641 | 1.6896 | 3.6403 | 2.7643 | 0.879 | 0.4463 | 4.5826 | 2.9989 | 2.378 | 1.551 | 0.7979 | 0.3487 | 1.3489 | 1.4148 | 0.9276 | 0.9022 |
| **Glo7421 (core)** | 3.669 | 4.1341 | 6.8158 | 8.1833 | 6.0132 | 7.6645 | 0.014 | 0.185 | 4.5279 | 5.6486 | 4.6537 | 6.1895 | 10.0712 | 9.658 | 7.719 | 7.6448 | 9.6839 | 9.8526 | 0.7711 | 1.1383 | 2.7783 | 3.9557 | 0.6463 | 1.1181 | 1.2084 | 2.1301 | 3.2977 | 4.7359 | 2.4765 | 2.2776 | 8.365 | 7.9573 |
| **Mic843  (core)** | 0.4924 | 0.4971 | 0.461 | 0.9066 | 0.2975 | 0.8087 | 4.5597 | 3.223 | 0.0186 | 0.4472 | 0.4579 | 0.6422 | 2.2482 | 2.179 | 1.5537 | 1.707 | 2.1479 | 2.1951 | 2.6545 | 1.9796 | 0.4151 | 0.2725 | 3.3313 | 2.0704 | 1.5234 | 1.0287 | 0.4245 | 0.309 | 0.8805 | 0.9245 | 1.3109 | 1.2611 |
| **Nos7120 (core)** | 0.5766 | 0.4637 | 0.6135 | 0.8692 | 0.5712 | 0.7888 | 4.6413 | 3.2135 | 0.4587 | 0.3971 | 0.0223 | 0.2698 | 1.7379 | 1.6341 | 0.9619 | 1.0596 | 1.6356 | 1.7042 | 2.7412 | 1.9011 | 0.6703 | 0.3609 | 3.3592 | 2.0485 | 1.4557 | 0.9143 | 0.6793 | 0.3382 | 1.0517 | 0.9216 | 0.8175 | 0.7776 |
| **ProMED4 (core)** | 2.5345 | 1.87 | 1.3466 | 0.6127 | 1.676 | 0.6188 | 10.074 | 7.847 | 2.2439 | 0.9684 | 1.7246 | 0.8041 | 0.0111 | 0.044 | 0.3253 | 0.322 | 0.0203 | 0.027 | 7.073 | 5.4771 | 3.5452 | 2.06 | 7.8441 | 5.5161 | 5.1235 | 3.542 | 3.3184 | 1.5554 | 4.0959 | 3.5931 | 0.5813 | 0.3705 |
| **Pro9211 (core)** | 1.4067 | 0.8979 | 0.9864 | 0.4775 | 1.1682 | 0.4197 | 7.7083 | 5.7843 | 1.5498 | 0.4676 | 0.9545 | 0.3329 | 0.3259 | 0.2816 | 0.0151 | 0.0624 | 0.2728 | 0.2938 | 5.1324 | 3.7595 | 2.3701 | 1.1555 | 5.7371 | 3.6805 | 3.4516 | 2.0773 | 2.2219 | 0.8306 | 2.6974 | 2.1991 | 0.3485 | 0.1991 |
| **Pro9215 (core)** | 2.4064 | 1.73 | 1.3299 | 0.6295 | 1.6293 | 0.6083 | 9.6602 | 7.485 | 2.1446 | 0.902 | 1.605 | 0.7384 | 0.0197 | 0.0363 | 0.2695 | 0.2658 | 0.0119 | 0.0239 | 6.802 | 5.2544 | 3.3759 | 1.9325 | 7.4989 | 5.2413 | 4.9447 | 3.4201 | 3.1746 | 1.4873 | 3.9296 | 3.4433 | 0.5835 | 0.3537 |
| **SycJA23 (core)** | 1.8291 | 2.2001 | 4.166 | 5.2993 | 3.638 | 4.9207 | 0.7678 | 0.4279 | 2.6346 | 3.3921 | 2.7375 | 3.8398 | 7.0493 | 6.8176 | 5.105 | 5.1053 | 6.8072 | 6.882 | 0.0167 | 0.1401 | 1.4401 | 2.1477 | 0.5059 | 0.478 | 0.6356 | 1.0188 | 1.6041 | 2.6619 | 1.0281 | 0.9218 | 5.5562 | 5.2615 |
| **Syc7002 (core)** | 0.4047 | 0.5938 | 1.2062 | 1.9583 | 0.8792 | 1.7677 | 2.7863 | 1.7601 | 0.4184 | 1.03 | 0.6618 | 1.2288 | 3.5462 | 3.4102 | 2.335 | 2.4895 | 3.3654 | 3.4559 | 1.439 | 1.0314 | 0.0195 | 0.2872 | 2.0109 | 1.1649 | 0.6856 | 0.5446 | 0.2012 | 0.5774 | 0.2853 | 0.3793 | 2.3177 | 2.2267 |
| **Syc7803 (core)** | 2.4616 | 2.8215 | 5.1695 | 6.1654 | 4.591 | 5.8165 | 0.6554 | 0.4379 | 3.3227 | 4.1575 | 3.3844 | 4.5376 | 7.8376 | 7.5046 | 5.6914 | 5.6578 | 7.5641 | 7.668 | 0.5011 | 0.6165 | 2.0554 | 2.8548 | 0.0192 | 0.3077 | 0.7773 | 1.279 | 2.366 | 3.4376 | 1.7588 | 1.4775 | 6.3668 | 6.0691 |
| **Syc7942 (core)** | 0.978 | 1.217 | 2.8367 | 3.651 | 2.3759 | 3.3267 | 1.1995 | 0.5698 | 1.5264 | 2.1151 | 1.4993 | 2.3826 | 5.1657 | 4.906 | 3.4569 | 3.5335 | 4.9204 | 5.0371 | 0.6342 | 0.409 | 0.6919 | 1.1576 | 0.7616 | 0.3483 | 0.0209 | 0.2862 | 1.0306 | 1.6009 | 0.6109 | 0.4723 | 3.7919 | 3.5857 |
| **Syn6803 (core)** | 0.3665 | 0.5688 | 1.0484 | 1.762 | 0.8059 | 1.5824 | 3.2846 | 2.1957 | 0.4271 | 0.9332 | 0.6788 | 1.1791 | 3.3286 | 3.183 | 2.1991 | 2.3363 | 3.1795 | 3.2352 | 1.6093 | 1.1554 | 0.206 | 0.3554 | 2.354 | 1.4152 | 1.0193 | 0.7679 | 0.0173 | 0.4104 | 0.3906 | 0.4996 | 2.1017 | 2.0141 |
| **ThermoBP1 (core)** | 0.4469 | 0.7065 | 1.7754 | 2.5061 | 1.3519 | 2.2319 | 2.4543 | 1.575 | 0.8667 | 1.3944 | 1.0564 | 1.646 | 4.1071 | 3.9425 | 2.7101 | 2.88 | 3.9235 | 3.974 | 1.0368 | 0.7039 | 0.2866 | 0.5396 | 1.7876 | 0.9557 | 0.6315 | 0.4694 | 0.3903 | 0.8368 | 0.0161 | 0.16 | 2.7486 | 2.6319 |
| **Trich101 (core)** | 1.4236 | 1.0284 | 0.6827 | 0.3005 | 0.9283 | 0.3185 | 8.373 | 6.4082 | 1.3034 | 0.5145 | 0.8192 | 0.2578 | 0.5831 | 0.5918 | 0.3421 | 0.4892 | 0.5784 | 0.5991 | 5.5395 | 4.176 | 2.3046 | 1.2257 | 6.3975 | 4.2845 | 3.8125 | 2.4391 | 2.1037 | 0.8525 | 2.7409 | 2.482 | 0.0193 | 0.1201 |

**Additional file S3:** **Table of differential codon usage.** This table shows the differences in the codon usage of core and unique genes compared across all 16 cyanobacterial strains. Each number indicates the difference in codon usage of the core genes of a particular species (row) and the core or unique genes of all species (columns) and is calculated as described in Materials and Methods. In short, we selected 100 different genes of one group of genes and estimated the relative abundance of each triplet for each amino acid. To compute the differences between two groups, we summarized the squared differences beween the rates of each codon.
