# Supplementary material for: The diversity of cyanobacterial metabolism: genome analysis of multiple phototrophic microorganisms
Source: BMC Genomics. 2012 Feb 2;13:56. doi: 10.1186/1471-2164-13-56 (PMC3369817; doi:10.1186/1471-2164-13-56)
Supplement: Additional file 5 — Differences in EC annotation. The pdf contains a list of EC numbers corresponding to groups A and D in Figure 7, respectively. [file 1471-2164-13-56-S5.PDF]

Additional File S7

The pdf contains a list of EC numbers corresponding to groups A and D in Figure 7, respectively.

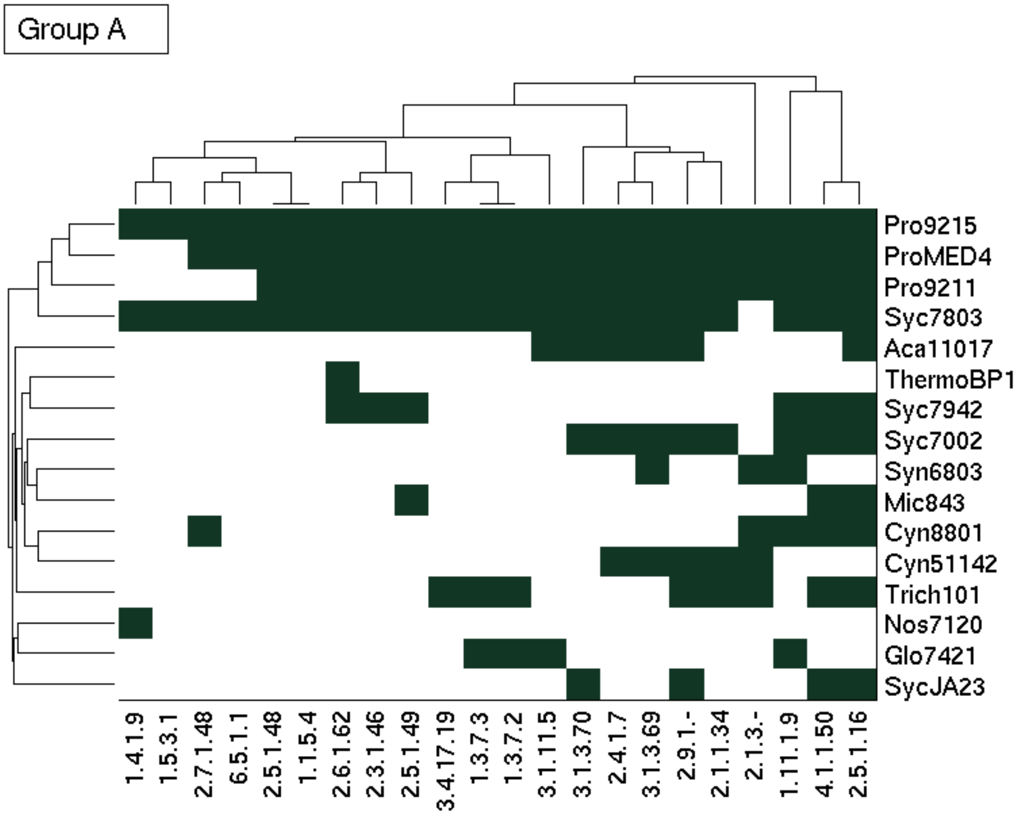

Group A contains EC numbers that are predominantely associated with the three Prochlorococcus strains (ProMED4, Pro9211, Pro9215) and the closely related Synechococcus sp. WH7803 (Syc7803). EC numbers exclusive to these four strains are a malate:quinone oxidoreductase (EC 1.1.5.4) involved in the TCA cycle and a cystathionine gamma-synthase (EC 2.5.1.48) which catalyzes one of the essential steps in the synthesis of the amino acid L-methionine. No EC numbers are exclusively annotated for the three Prochlorococcus strains (ProMED4, Pro9211, Pro9215).

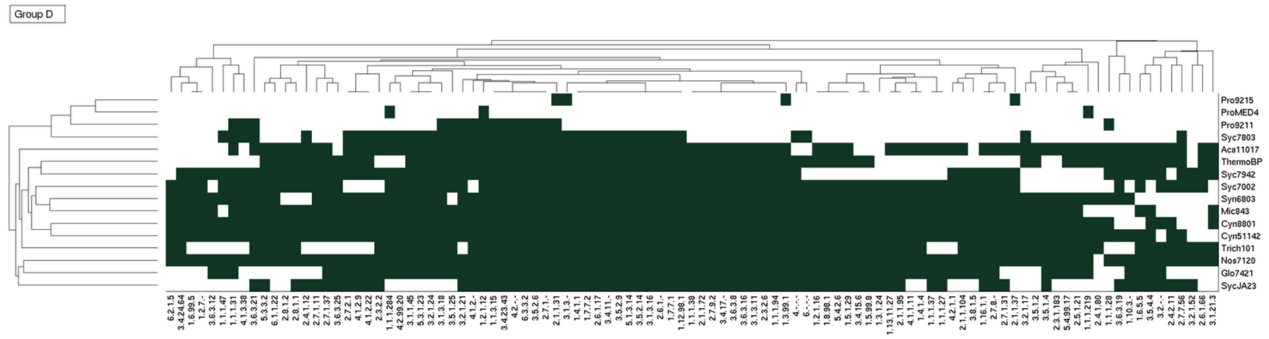

Group D contains EC numbers that are annotated for almost all strains, except the three Prochlorococcus strains (ProMED4, Pro9211, Pro9215) and the closely related Synechococcus sp. WH7803 (Syc7803). Annotated EC numbers for all strains, except the four strains mentioned above correspond to a malate dehydrogenase (EC 1.1.1.94), a site-specific DNA-methyltransferase (EC 2.1.1.72), a leucyl,phenylalanine-tRNA-protein transferase (EC 2.3.2.6), a pyruvate water dikinase (EC 3.1.3.11), a fructose-1,6-bisphosphatase (EC 3.1.3.11), an unspecific carboxypeptidase (EC 3.4.17.-), and the transporters (EC 3.6.3.16) for arsenite and (EC 3.6.3.8) for calcium.

Annotated EC numbers for all strains, except the three Prochlorococcus strains correspond to a coenzyme F420 hydrogenase (EC 1.12.98.1), an alanine dehydrogenase (EC 1.4.1.1), a ferredoxin dependent nitrite and nitrate reductase (EC 1.7.7.1 and EC 1.7.7.2), an alanine-glyoxylate aminotransferase 2-like (EC 2.6.1.-), a succinyldiaminopimelate transaminase (EC 2.6.1.17), a protein phosphatase (EC 3.1.3.16), an aminopeptidase (EC 3.4.11.-), a N-methylhydantoin amidohydrolase (EC 3.5.2.14), a pyroglutamate hydrolase (EC 3.5.2.9), and an UDP-N-acetylglucosamine 2-epimerase (EC 5.1.3.14).
