# Supplementary material for: The diversity of cyanobacterial metabolism: genome analysis of multiple phototrophic microorganisms
Source: BMC Genomics. 2012 Feb 2;13:56. doi: 10.1186/1471-2164-13-56 (PMC3369817; doi:10.1186/1471-2164-13-56)
Supplement: Additional file 7 — Annotated phylogenetic tree. Shown is a maximum-likelihood distance tree for sequenced cyanobacteria reproduced from Gupta et al. 2010 [9]. Strains chosen for analysis are indicated by red arrows. [file 1471-2164-13-56-S7.PDF]

Additional file S6

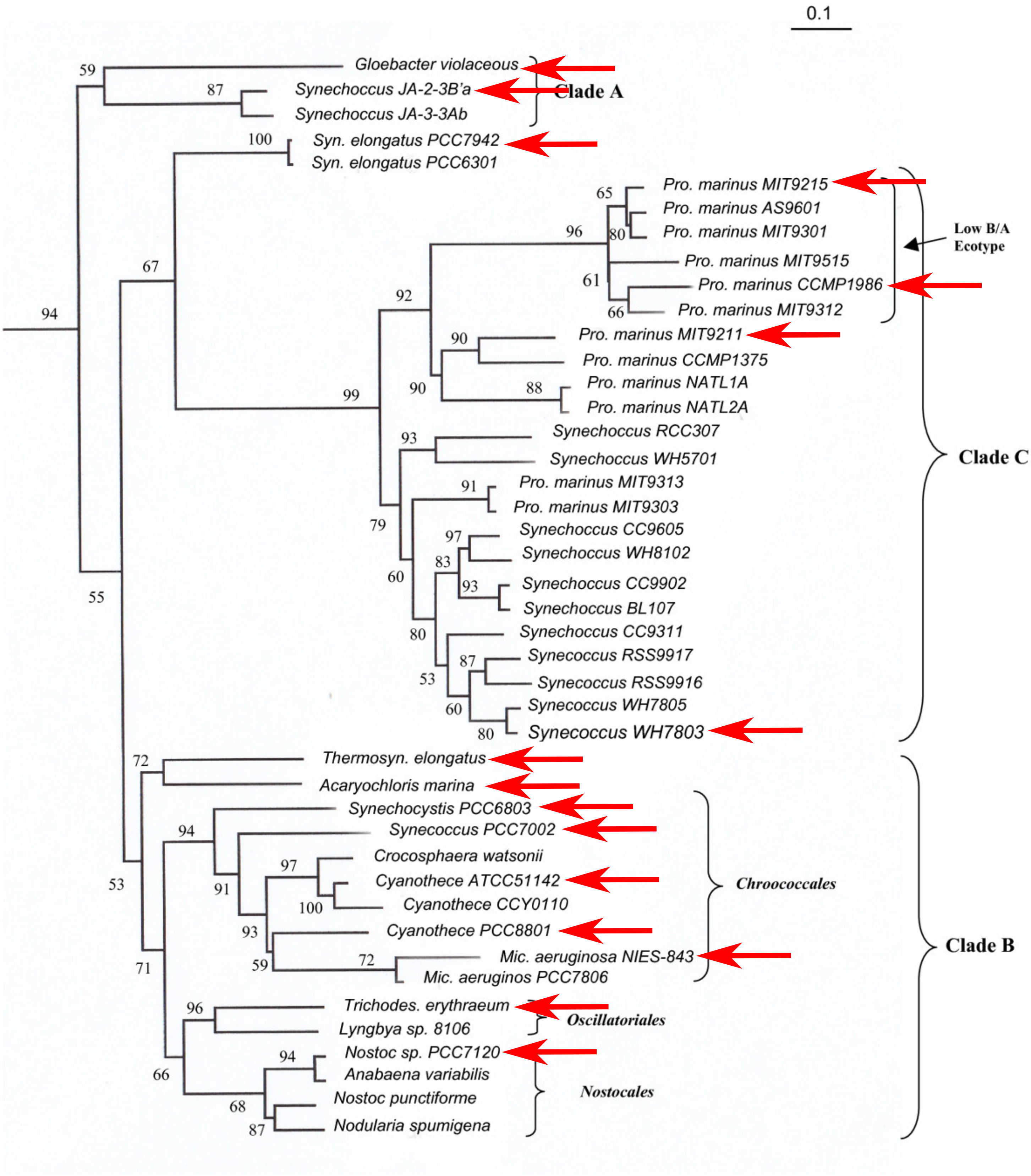

Selection of cyanobacterial strains across different Clades.

Shown is a maximum-likelihood distance tree for sequenced cyanobacteria reproduced from Gupta et al. 2010. Strains chosen foranalysis are indicated by red arrows. Our aim was to obtain areasonable representation of all three Clades (A-C) defined by Gupta et al. 2010.
